# Identification and characterization of microRNAs from Chinese pollination constant non-astringent persimmon using high-throughput sequencing

**DOI:** 10.1186/s12870-014-0400-6

**Published:** 2015-01-21

**Authors:** Yujie Luo, Xiaona Zhang, Zhengrong Luo, Qinglin Zhang, Jihong Liu

**Affiliations:** Key Laboratory of Horticultural Plant Biology (MOE), College of Horticulture and Forestry Science, Huazhong Agricultural University, Wuhan, 430070 China

**Keywords:** *Diospyros kaki* Thunb, Deastringency, High-throughput sequencing, MicroRNA, Proanthocyanidins, Target identification

## Abstract

**Background:**

microRNAs (miRNAs) have been shown to play key roles in regulating gene expression at post-transcriptional level, but miRNAs associated with natural deastringency of Chinese pollination-constant nonastringent persimmon (CPCNA) have never been identified.

**Results:**

In this study, two small RNA libraries established using ‘Eshi No. 1’ persimmon (*Diospyros kaki* Thunb.; CPCNA) fruits collected at 15 and 20 weeks after flowering (WAF) were sequenced through Solexa platform in order to identify miRNAs involved in deastringency of persimmon. A total of 6,258,487 and 7,634,169 reads were generated for the libraries at 15 and 20 WAF, respectively. Based on sequence similarity and hairpin structure prediction, 236 known miRNAs belonging to 65 miRNA families and 33 novel miRNAs were identified using persimmon transcriptome data. Sixty one of the characterized miRNAs exhibited pronounced difference in the expression levels between 15 and 20 WAF, 17 up-regulated and 44 down-regulated. Expression profiles of 12 conserved and 10 novel miRNAs were validated by stem loop qRT-PCR. A total of 198 target genes were predicted for the differentially expressed miRNAs, including several genes that have been reported to be implicated in proanthocyanidins (PAs, or called tannin) accumulation. In addition, two transcription factors, a GRF and a bHLH, were experimentally confirmed as the targets of dka-miR396 and dka-miR395, respectively.

**Conclusions:**

Taken together, the present data unraveled several important miRNAs in persimmon. Among them, miR395p-3p and miR858b may regulate bHLH and MYB, respectively, which are influenced by SPL under the control of miR156j-5p and in turn regulate the structural genes involved in PA biosynthesis. In addition, dka-miR396g and miR2911a may regulate their target genes associated with glucosylation and insolubilization of tannin precursors. All of these miRNAs might play key roles in the regulation of (de)astringency in persimmon fruits under normal development conditions.

**Electronic supplementary material:**

The online version of this article (doi:10.1186/s12870-014-0400-6) contains supplementary material, which is available to authorized users.

## Background

Oriental persimmon (*Diospyros kaki* Thunb. 2n = 6× = 90) is distributed in the mountainous areas adjacent to the three provinces, Hubei, Henan and Anhui, of central China [1]. According to the criteria established for persimmon cultivars, persimmon can be categorized into two major groups, pollination-constant nonastringent (PCNA) type consisting of two subcategories, Chinese PCNA (CPCNA) and Japanese PCNA (J-PCNA), non-PCNA type consisting of three subcategories, pollination-constant astringent (PCA), pollination-variant nonastringent (PVNA), and pollination-variant astringent (PVA) [2]. The CPCNA-type fruits are able to lose astringency naturally at ripening stage, thus justifying their significant value as commercial use. Non-astringency is a discrete trait for the CPCNA fruits, but is a quantitative trait for non-PCNA fruits. The genetic trait of CPCNA has been shown to be controlled by a single locus (*CHINESE PCNA*, denoted as *CPCNA*), which is dominant against JPCNA [3,4], implying that one half of the F_1_ offspring derived from crosses between CPCNA and JPCNA will generate PCNA-type fruits [3,5]. Therefore, persimmon CPCNA cultivars hold great potential for breeding new cultivars of PCNA type. However, limited information is available on the molecular mechanism underlying fruit (de)astringency of CPCNA persimmon. Therefore, elucidation of the molecular mechanisms underlying natural loss of fruit astringency in CPCNA persimmon is of paramount significance for persimmon genetic improvement.

Astringency of persimmon fruits is ascribed to the accumulation of tannins (proanthocyanidins, PAs), which are biosynthesized via three main pathways through shikimate, flavonoid and PA [6]. A majority of genes in these pathways have been isolated, including *PAL*, *CHS*, *CHI*, *F3H*, *F3′5′H*, *DFR*, *ANS*, *LAR*, and *ANR*. Expression patterns of these genes were analyzed in fruits of CPCNA and JPCN persimmon, which showed that transcript levels of most genes were lower in JPCNA than in CPCNA from middle to late developmental stages [7]. In addition, *DkPDC* and *DkADH* were suggested to be associated with natural astringency loss of CPCNA persimmon [8]. Meanwhile, great progresses have also been achieved regarding elucidation of transcriptional regulation in recent years. For example, a basic helix-loop-helix (bHLH) transcription factor (TF), DkMYC1, was isolated from ‘Luotian-tianshi’, a famous CPCNA, which is proposed to control PA biosynthesis by regulating expression of *DkLAR* and *DkANR* through binding to relevant *cis*-elements on the gene promoters [9]. In another work, genome-wide transcriptome analysis of CPCNA identified a number of TFs associated with PA biosynthesis, including 12 MYBs, three bHLHs and two WD40s [10]. The PA monomers are transported to vacuoles through TT12 and TT19, and then polymerized into polymeric PAs catalyzed by LAC [11,12]. However, it is worth mentioning that despite above-mentioned work on elucidation of proanthocyanidin biosynthesis, the underlying molecular mechanisms of natural astringency loss remain largely elusive, and further in-depth analyses are required to dissect the mechanisms.

Apart from transcriptional regulation, post-transcriptional regulation by microRNAs (miRNAs) is also crucial for a number of physiological processes. The miRNAs are a class of endogenous non-coding small RNAs of 20–24 nucleotides (nt). The biogenesis of miRNA has been well documented. First, long single-stranded primary miRNAs (pri-miRNAs) are generated from the intragenic regions of nuclear-encoded *MIR* genes by RNA polymerase II [13-15]. Then, the pri-miRNAs are transcribed in nucleus to generate 100–200 nt precursor miRNAs (pre-miRNAs) with stem-loop structures (hairpins) catalyzed by Dicer-like I enzyme (DCL1), yielding a duplex intermediate (miRNA/miRNA*) [16-18]. After addition of a 5′ 7-methylguanosine cap by HuaEnhancer1 (HEN1) [19], the RNA duplexes are translocated into cytoplasm by HASTY, a plant protein orthologous to exportin-5 [20]. Finally, the mature miRNA strand is integrated with RNA-induced silencing complex (RISC), whereas the miRNA^*^ strand is usually degraded [21]. The RISC is then incorporated with AGRONAUTE proteins (AGO) and functions to regulate target gene expression through cleaving the target mRNA, leading to repression of mRNA translation [22]. In plants, most miRNAs can perfectly complement with their mRNA targets, while the single recognition site is predominantly present in the mRNA coding region rather than in the 3-untranslated region (UTR) [13]. Plant miRNAs have been predicted or validated to regulate genes encoding various types of proteins that play pivotal roles in many biological processes [23].

Currently, two main approaches are usually applied to study miRNAs, computational prediction using ESTs or genomic sequences and next generation sequencing-based techniques [20,24-26]. Given that the computation-based approach is restricted to discovering conserved miRNAs and that genomic information of persimmon is scarce thus far, the second approach may be more suitable for deciphering miRNAs in persimmon. In this study, deep sequencing using Illumina GA_II_ was applied to identify both conserved and novel miRNAs that are possibly implicated in fruit (de)astringency of ‘Eshi No. 1’ persimmon (*Diospyros kaki* Thunb.). Stem-loop quantitative real-time RT-PCR (qRT-PCR) [27] was employed to validate the expression level of a set of miRNAs. In addition, identification and characterization of miRNAs and their target genes were established using bioinformatics prediction in combination with 5′-RACE.

## Results

### Determination of PA contents in persimmon fruits

**Figure 1:**
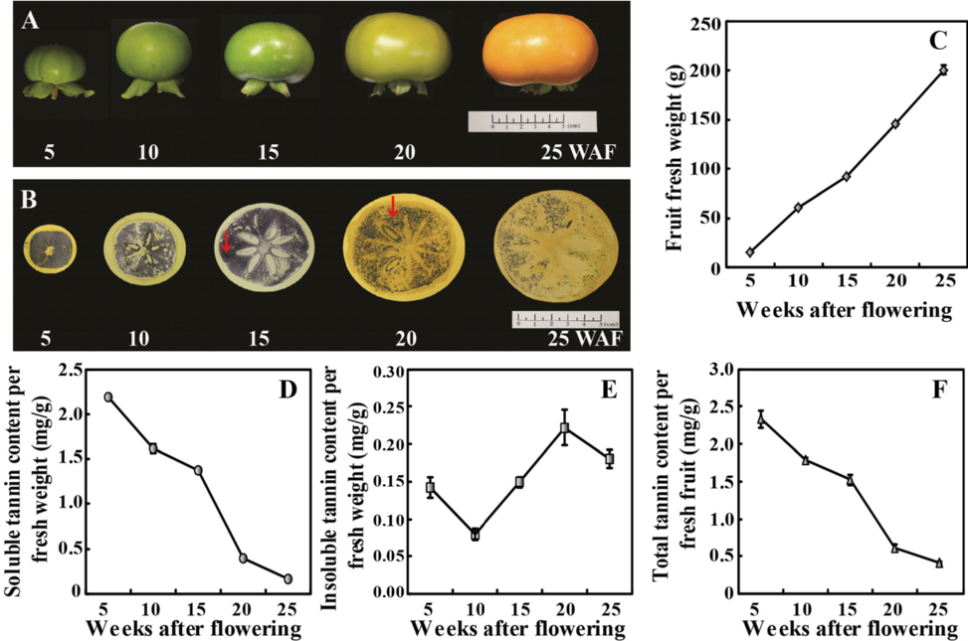
**Measurement of tannin content in ‘Eshi No. 1’ (CPCNA) fruits at different development stages. A**. Representative photos showing the fruits sampled at five stages, 5, 10, 15, 20 and 25 weeks after flowering (WAF) of ‘Eshi No. 1’. **B**. Analysis of soluble tannin content in the persimmon fruits based on an imprinting method. The red arrows show that the staining became weaker from 15 to 20 WAF. **C**. Change in the fruit weight at the five sampling stages. **D-E**. Quantitative measurement of soluble **(D)** and insoluble **(E)** tannin in the fruits by folin-ciocalteu method. **F**. Total tannin content in the fruits.

Imprinting method was used to determine soluble tannin levels in ‘Eshi No. 1’ fruits. The sections were deeply stained at the beginning of fruit development (5 WAF), when the fruits were small in size. With the progression of development, the fruits grew quickly and became increasingly big until reaching the largest size at 25 WAF (Figure 1A,C). The fruits were still darkly stained until 15 WAF, but the staining began to turn lighter at 20 WAF. At the last experimental stage, 25 WAF, the fruits were only slightly stained (Figure 1B).

To confirm the imprinting results, quantitative measurement of soluble and insoluble tannin contents in the fruits was carried out using the Folin-Ciocalteu method. The soluble tannin in the fruits was 2.19 mg/g FW at 5 WAF, but quickly decreased at 10 WAF (1.61 mg/g FW), followed by a slight change at 15 WAF. However, a sharp decrease of soluble tannin in the fruits was observed between 15 and 20 WAF, changing from 1.37 to 0.39 mg/g FW. The tannin level in the fruits at 25 WAF (0.17 mg/g FW) was only slighted decreased compared with that of 20 WAF (Figure 1D). At the last stage, soluble tannin accounts for less than 0.2% of the fruit weight, implying that the fruits at this point have already lost their astringency [28]. The insoluble tannin, remarkably less than the soluble tannin, was decreased to the lowest level (0.08 mg/g FW) from 5 to 10 WAF, but progressively increased thereafter, reaching the peak value (0.22 mg/g FW) at 20 WAF, followed by a minor decrease at 25 WAF (Figure 1E). Total tannin contents in the fruits followed the trend of soluble type during the whole developmental stage (Figure 1F).

As the soluble tannin underwent the greatest change between 15 and 20 WAF, and the insoluble tannin increased to the largest amount during this stage, the fruits at these two stages were selected for miRNA sequencing in the subsequent work.

### Sequencing of small RNA libraries using Illumina platform

To identify persimmon miRNAs, two small RNA (sRNA) libraries were constructed using fruits collected at 15 and 20 WAF, and subjected to deep sequencing. A total of 6,258,487 and 7,634,169 raw reads were obtained at 15 and 20 WAF, respectively. After removing low quality sequences, adapters, poly-A sequences and small sequences shorter than 12 nt, 6,091,310 (15 WAF) and 7,442,012 (20 WAF) clean reads and 2,348,888 (15 WAF) and 1,970,898 (20 WAF) unique sequences were finally generated (Table 1). The sRNA data have been deposited in NCBI (National Center for Biotechnology Information), under the accession number of SRP050516.

**Table 1:** **Summary of small RNA sequencing in** ***Diospyros kaki*** **Thunb. small RNA libraries constructed using fruits collected at 15 and 20 weeks after flowering (WAF)**

**Libraries**	**Raw reads**	**Clean reads**	**Unique reads**	**Redundant reads**
15 WAF	6,258,487	6,091,310 (97.33%)	2,348,888 (38.48%)	3,747,422 (61.52%)
20 WAF	7,634,169	7,442,012 (97.48%)	1,970,898 (26.48%)	5,471,114 (73.52%)

**Figure 2:**
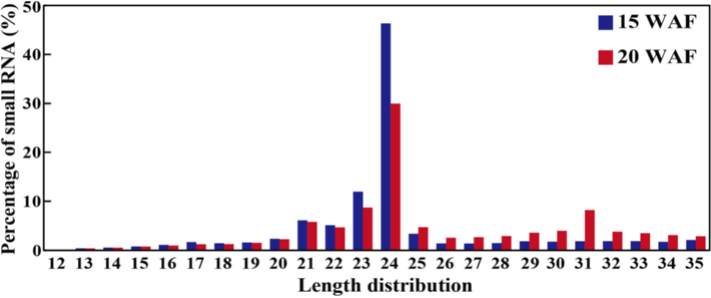
**Length distribution of small RNAs in the two libraries constructed using fruits sampled at 15 and 20 weeks after flowering (WAF) of ‘Eshi No. 1’.**

As composition of small RNAs reflects their different roles in specific functions [29], we investigated length distribution of the small RNAs in the two libraries. The results demonstrated that the majority of miRNAs ranged from 20 to 25 nt in length, in which small RNAs of 24 nt were most abundant in the two libraries, accounting for 37.1% and 23.2%, respectively (Figure 2). In order to get a clear view of sequence annotation, the small RNA reads were searched against Rfam 11.0 (http://rfam.sanger.ac.uk/) database, which revealed that 39.4% and 18.3% of the sequences at 15 WAF and 20 WAF, respectively, can be annotated to non-coding small RNAs (rRNAs, tRNAs, siRNAs, snRNAs, snoRNAs, miRNAs, unknown sRNAs). However, only 1.8% and 1.5% of the miRNAs accounting for the total sRNAs were identified in the two libraries (Table 2).

**Table 2:** **The annotation of number and percentage (in parenthesis) of various components (rRNA, snRNA, snoRNA, tRNA, miRNA, and others) in** ***Diospyros kaki*** **Thunb. small RNA libraries constructed using fruits collected at 15 and 20 weeks after flowering (WAF)**

**Category**	**Libraries**	
	**15 WAF**	**20 WAF**
rRNAs	1,648,954 (27.07%)	1,067,675 (14.35%)
snRNAs	17,709 (0.29%)	16,534 (0.22%)
snoRNAs	35,937 (0.59%)	31,779 (0.43%)
tRNAs	583,076 (9.57%)	132,709 (1.78%)
miRNA	111,535 (1.83%)	109,566 (1.47%)
Others	3,692,129 (60.61%)	6,082,268 (81.73%)
Total	6,091,310 (100%)	7,442,012 (100%)

### Identification of known and novel miRNAs

**Figure 3:**
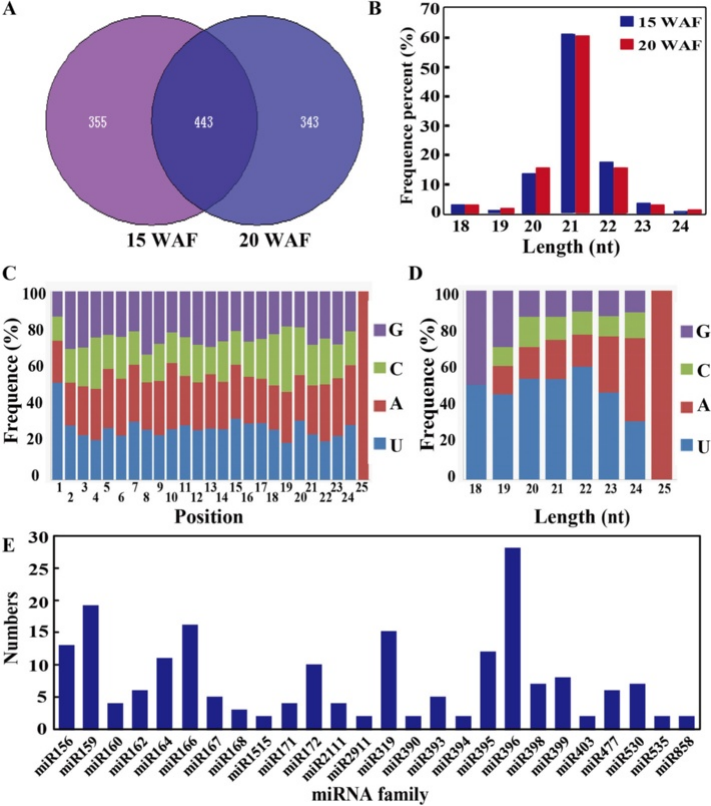
**Identification of known miRNAs in the two small RNA libraries of ‘Eshi No. 1’. A**. The number of known miRNAs in the two libraries constructed using fruits collected at 15 and 20 weeks after flowering (WAF). **B**. Proportion of known miRNAs with different length in the two libraries. **C**. Nucleotide preference at each position of the known miRNAs. **D**. Analysis of first nucleotide bias in the miRNAs of different length. **E**. The number of miRNA members in the 26 families with more than one member.

The sequences were searched against miRBase v21.0, in which miRNAs from 73 plant species have been deposited [30]. After alignment, a total of 1,141 miRNAs were obtained, in which 355 and 343 miRNAs were unique to the 15 WAF and 20 WAF libraries, respectively, while 443 miRNAs were present in both libraries (Figure 3A, Additional file 1: Table S1). Length distribution analysis showed that most of the known miRNAs were clustered in the 21-nt type (Figure 3B). We then analyzed nucleotide bias at each position so as to understand whether the cleavage sites of miRNAs had specific features for miRNA [31]. About 51.4% of the miRNAs had uridine (U) at their first nucleotide position, but resistance to guanine (G) was observed at the first position. By contrast, positions between 2 and 4 were resistant to U. We also found that the tenth nucleotide, a position determining the cleavage site, had a strong preference for adenosine (A) (Figure 3C). Due to different cleavage sites of DCL enzymes and some other factors, additional or missing nucleotides may exist at the end of mature miRNAs, especially at the 5′ end [32]. We also noticed that different from 21-nt miRNAs, the first position of 24-nt miRNAs showed a strong preference for A (Figure 3D).

The small RNAs with at least seven reads in one of the two libraries were used to search the database, which gave rise to 236 known miRNAs in the two libraries. After family analysis, these known miRNAs were found to belong to 65 miRNA families, in which 39 families have one member. The largest miRNA family is miR396 composed of 28 members, followed by miR159 with 19 members (Figure 3E, Additional file 1: Table S1).

Expression analysis was performed based on normalized read counts for each miRNA family. It showed that miRNA abundance was different among the 65 known families. The highly conserved miRNAs, such as miR156/miR157, miR159, miR160, miR166 and miR319, were expressed abundantly. The most abundant miRNAs were miR396 and miR162, with 80,686 and 52,111 TPM (transcripts per million) in the two libraries, respectively. However, non-conserved miRNAs, such as miR535, miR167, miR2275, miR530 and miR418, were expressed at relatively lower levels (Additional file 1: Table S1).

Based on the criteria for selecting differentially expressed miRNAs, including |fold change| > 1 and *P*-value < 0.05, 61 out of the 236 known miRNAs were identified to exhibit different expression levels between 15 and 20 WAF. Among the 61 differentially expressed miRNAs, 17 were up-regulated, whereas 44 were down-regulated, in the fruits at 20 WAF in comparison with those at 15 WAF (Table 3), in which 33 showed a two-fold or greater (ratio > 2 and *P*-value < 0.05) change. Of note, the members in families of dka-miR160, dka-miR398, dka-miR535, and dka-miR827 were only up-regulated, whereas those of dka-miR159, dka-miR164, dka-miR2111, dka-miR395, dka-miR396, dka-miR399, dka-miR530, and dka-miR858 were all down-regulated.

**Table 3:** **Differentially expressed miRNAs in the two small RNA libraries constructed using fruits collected at 15 and 20 weeks after flowering (WAF), based on transcripts per million (TPM) and fold change (FC) at** ***P*** **-value < 0.05**

**Names**	**Sequences**	**TPM**		***P*** **-value**	**FC**
		**15 WAF**	**20 WAF**		
dka-miR156a	CGACAGAAGAGAGTGAGCAC	0.010	1.075	0.008	6.748
dka-miR156d	TGACAGAAGAGAGTGAGCACA	34.475	322.359	0.000	3.225
dka-miR156j	GTTGACAGAAGAGAGTGAGCAC	0.657	11.556	0.008	4.137
dka-miR156j-5p	TGACAGAAGAGAGTGAGCAC	65.011	1384.572	0.000	4.413
dka-miR156s	TGACAGAAGAGAGTGAGCACT	0.657	9.137	0.006	3.799
dka-miR160a	TGCCTGGCTCCCTGAATGCCA	0.010	1.344	0.000	7.070
dka-miR167c	TGAAGCTGCCAGCATGATCTGG	0.657	1.612	0.000	1.296
dka-miR167g	TGAAGCTGCCAGCATGATCTGA	0.010	1.344	0.000	7.070
dka-miR171n-3p	TGATTGAGCCGCGCCAATATC	0.010	1.344	0.001	7.070
dka-miR2275c	AGAATTGGAGGAAAACAAACTGA	0.010	1.344	0.001	7.070
dka-miR2911a	GGCCGGGGGACGGACTGGGA	158.587	890.888	0.001	2.490
dka-miR398a	TGTGTTCTCAGGTCACCCCTT	10.507	1868.581	0.000	7.474
dka-miR398b	TGTGTTCTCAGGTCACCCCT	0.010	1.209	0.000	6.918
dka-miR398c	TGTGTTCTCAGGTCACCCCTG	0.010	1.478	0.000	7.208
dka-miR398d	TGTGTTCTCAGGTCACCCCTC	0.010	1.209	0.000	6.918
dka-miR535c	TGACAAGGAGAGAGAGCACGC	0.010	3.494	0.000	8.449
dka-miR827-3p	TTAGATGACCATCAGCAAACA	0.010	3.225	0.000	8.333
dka-miR159a-5p	GAGCTCCTTGAAGTCCAATTG	6.238	2.150	0.000	−1.537
dka-miR159e-5p	GAGCTCCTTGAAGTCCAATT	5.418	1.612	0.000	−1.748
dka-miR164d	TGGAGAAGCAGGGCACGTGC	9.358	4.031	0.003	−1.215
dka-miR164e	TGGAGAAGCAGGGCACGTGCG	51.056	21.231	0.001	−1.266
dka-miR164f-5p	TGGAGAAGCAGGGCACGTGCT	46.624	18.543	0.000	−1.330
dka-miR164h-3p	CATGTGCCCTTCTTCTCCATC	3.940	1.344	0.000	−1.552
dka-miR167a	TGAAGCTGCCAGCATGATCTC	1.642	0.010	0.008	−7.359
dka-miR171i	CGAGCCGAATCAATATCACTC	49.250	18.946	0.000	−1.378
dka-miR172g-5p	GGAGCATCATCAAGATTCACA	3.283	0.537	0.004	−2.611
dka-miR2111a	TAATCTGCATCCTGAGGTTTA	30.207	7.525	0.004	−2.005
dka-miR2111d	GTCCTTAGGATGCAGATTACC	185.346	32.921	0.000	−2.493
dka-miR2916	TGGGGACTCGAAGACGATCATAT	7.880	2.553	0.000	−1.626
dka-miR319i	TTGGACTGAAGGGTGCTCCCT	11.820	4.837	0.000	−1.289
dka-miR390b-3p	CGCTATCTATCCTGAGCTCCA	1.313	0.537	0.001	−1.289
dka-miR395f	ATGAAGTGTTTGGGGGAACTC	16.745	6.853	0.001	−1.289
dka-miR395p-3p	GTGAAGTGTTTGGGGGAACTC	37.266	16.125	0.000	−1.209
dka-miR396a-3p	GTTCAAGAAAGCTGTGGGAAA	168.601	49.852	0.003	−1.758
dka-miR396a	CACAGCTTTCTTGAACTTTCT	7.223	1.747	0.000	−2.048
dka-miR396b	TTCCACAGCTTTCTTGAACT	43.012	17.334	0.000	−1.311
dka-miR396d	CTCCACAGGCTTTCTTGAACTG	43.012	18.140	0.002	−1.246
dka-miR156l-5p	TGACAGAAGAGAGAGAGCACA	1.480	0.010	0.000	−7.210
dka-miR396f	TTCCACGGCTTTCTTGAACTG	5.418	0.010	0.000	−9.081
dka-miR396f-3p	GGTCAAGAAAGCTGTGGGAAG	365.767	125.772	0.000	−1.540
dka-miR396f-5p	TCTCCACAGGCTTTCTTGAACT	6.567	2.553	0.000	−1.363
dka-miR396g	TTCCACAGCTTTCTTGAACTT	62326.659	26262.521	0.000	−1.247
dka-miR396i-3p	GTTCAATAAAGCTGTGGGAAG	507.937	213.383	0.000	−1.251
dka-miR396k	TTCCACGGCTTTCTTGAACC	7.388	2.956	0.002	−1.321
dka-miR396m	TTCCACAGCTTTCTTGAACTA	217.851	87.879	0.000	−1.310
dka-miR396r	TTCCACGGCTTTCTTGAACTT	16.253	5.509	0.000	−1.561
dka-miR396s	TCCCACAGCTTTATTGAACTG	5.910	1.747	0.000	−1.758
dka-miR399a	TGCCAAAGGAGAATTGCCC	2.627	0.010	0.000	−8.037
dka-miR399b	TGCCAAAGGAGAATTGCCCGG	2.627	0.010	0.002	−8.037
dka-miR399c	TGCCAAAGGAGAGTTGCCCTA	6.238	2.419	0.008	−1.367
dka-miR399f	TGCCAAAGGAGAATTGCAC	2.463	0.010	0.000	−7.944
dka-miR399h	TGCCAAAGGAGATTTGCCCCG	4.925	1.075	0.000	−2.196
dka-miR399h-3p	TGCCAAAGGAGAATTGCCCTG	135.275	35.474	0.000	−1.931
dka-miR479	CGTGATATTGGTTCGGCTCATC	31.192	10.481	0.000	−1.573
dka-miR530a	TGCATTTGCACCTGCACCTC	1.149	0.010	0.000	−6.844
dka-miR530b	TGCATTTGCACCTGCACCTT	7.059	0.010	0.000	−9.463
dka-miR530c	TGCATTTGCACCTGCACCTTG	1.313	0.010	0.000	−7.037
dka-miR530d	TGCATTTGCACCTGCACTTTC	1.970	0.010	0.000	−7.622
dka-miR530e	TGCATTTGCACCTGCACTTTA	1.313	0.010	0.002	−7.037
dka-miR530f	TGCATTTGCACCTGCATCTC	1.970	0.010	0.002	−7.622
dka-miR858b	TTCGTTGTCTGTTCGACCTTG	36.938	11.690	0.004	−1.660

After excluding known miRNAs and Rfam annotation, the remaining sequences were used to discover novel and potential persimmon-specific miRNAs, which revealed that a total of 33 miRNAs were predicted to be potentially novel. Secondary fold structures of precursors for the 33 novel miRNAs were analyzed (Additional file 2: Figure S1). Seven out of the 33 miRNAs were shown to have complementary miRNA* sequences (Table 4). In order to test the reliability of novel miRNA prediction, five novel miRNAs (miRN12, miRN15, miRN16, miRN25, and miRN31) were amplified and sequenced, in which four were completely consistent with those of deep sequencing, while only miRN15 was slightly different due to insertion of one nucleotide (Additional file 2: Figure S1). Most of the novel miRNAs were found to exist at low copies, with the exception of dka-miRN14, dka-miRN07, dka-miRN19, dka-miRN27, dka-miRN28, which possessed more than 1,000 reads (Table 4). Furthermore, 27 novel miRNAs were shown to be differentially expressed during fruit development, in which 10 were up-regulated, but 17 were down-regulated, at 20 WAF (Table 4).

**Table 4:** **Novel miRNA in the** ***Diospyros kaki*** **small RNA libraries constructed using fruits collected at 15 and 20 weeks after flowering (WAF)**

**Names**	**Sequences**	**Transcriptome ID**	**Score of minimal free energy (MFE)**	**15 WAF**	**20 WAF**	miRNA*	**Star sequences**	**Fold change**
dka-miRN01	aacccgaaauucgagaaagac	MGB_c3657_6185	1.2	20	0	No	ucguucgauuucgggucuggc	−8.36
dka-miRN02	aagaccugagaaugcaggaaagcuu	MGB_lrc11318_15117	1.8	10	0	No	gcugcugcacuuaaggucggaa	−7.36
dka-miRN03	aagucuaugauaagauggcugagu	MGB_c832_1961	0.2	196	135	No	gcaucuaguuauuguacaacuugc	−0.83
dka-miRN04	aaguggugcuuggaggaacuc	MGB_s80448_44080	1.7	0	15	No	gauucucgagugccagaagg	7.66
dka-miRN05	acggaguagaagccggccgagaau	MGB_s66037_35157	1.7	10	0	No	caucguccggcuucuccaccgaga	−7.36
dka-miRN06	acuuuacaaauaaagcccgaggau	MGB_lrc56264_28052	0.1	0	10	No	cuuugggcuuugauuaccaaggaa	7.07
dka-miRN07	agggugcggguuugggacagcc	MGB_lrc64120_34018	2	2220	1935	No	guguuugauccgcacccuuu	−0.49
dka-miRN08	aguacuugaggagaaggaaca	MGB_c17696_19188	0.8	14	0	No	auugauuucuggaaguaguag	−7.84
dka-miRN09	caauaaauucuagaauuuuggagu	MGB_c35059_23284	4.9	324	175	Yes	ccaagauuuagagacuuuaaagau	−1.18
dka-miRN10	cagaguacuacacuagcaacgcaua	MGB_c5218_8307	2	10	0	No	uguguguuuguguugggcuuuggg	−7.36
dka-miRN11	ccuaaauuagugauggcuuagggc	MGB_c45699_25796	1.3	40	30	No	ccuugagccgcuccugnunungagg	−0.70
dka-miRN12	auguaacagucacaugguagu	MGB_c66734_31898	1.8	0	20	No	uaccaugugauugucacauga	8.07
dka-miRN13	cggccgaaucguuggaugcgcc	MGB_c71082_39230	1.3	25	10	Yes	ucuacauuauucggcuauagu	−1.61
dka-miRN14	cuacaugaauucuagacuucuaug	MGB_c80774_44313	1.2	12780	0	Yes	uuugucuagaauucauauuuggau	−17.67
dka-miRN15	caugucaacuaaguuauggaaca	MGB_lrc74843_39335	8.5	350	0	Yes	uuuccauauuguacuuguugg	−12.49
dka-miRN16	cccguagguuauaggguuggcaac	MGB_c1713_3178	2.2	0	142	No	cuuuaaccugaacuuaccuuggcc	10.90
dka-miRN17	cuccuaacucuugcccauuuau	MGB_lrc49089_29889	3.1	10	0	No	aaaugggcaagaguuaggagag	−7.36
dka-miRN18	gagauaccagggaguagcagc	MGB_c8777_12556	2.7	10	0	No	aaguagauccaucggauu	−7.36
dka-miRN19	gauucaagaaagcuguggaau	MGB_lrc36442_25847	2.2	1152	988	Yes	uccacaggcuuucuugaacugc	−0.51
dka-miRN20	gggaauguacggaucuguaagua	MGB_c69353_37788	1.9	40	0	No	cuugcgauuugugcuucguuu	−9.36
dka-miRN21	ggucaauggucagugacuaggagu	MGB_c36458_25861	1.6	95	0	No	ucuaacgucaccaaacaguugaccc	−10.61
dka-miRN22	gugaauauugcagaccagcgg	MGB_lrc29703_21994	2.4	0	10	No	ucuggucugcaauauucaccc	7.07
dka-miRN23	ggagaugccagggagcagcgc	MGB_c6581_10173	2.7	0	211	No	gaugcucccucguaucuccau	11.46
dka-miRN24	guuggggauccauucgacuggggu	MGB_c8988_13431	1.7	0	10	No	uucaguugggguuuuucgauug	7.07
dka-miRN25	gguaugcauuccgcuguaggacc	MGB_c59771_28802	2.5	0	25	No	ucuuacagcagaaugcauacugc	8.39
dka-miRN26	guuugugaccaggggauuccc	MGB_c68424_33583	1.4	0	15	No	uuaucacuacugggcccauaagucu	7.66
dka-miRN27	uaaauccgagggaucuacuuu	MGB_c74194_41683	2.3	6404	5852	Yes	aaguagauccaucggauu	−0.42
dka-miRN28	ucuacuuuauagaacaccggc	MGB_c43859_28278	1.6	1349	0	No	uguuuucuauaaaguaaauc	14.43
dka-miRN29	auagcgguccuacagcgguaugca	MGB_c17398_18816	2.2	0	8	No	auuccgcuguagacccgugagcuc	7.17
dka-miRN30	ugagaaccaugugaaucacu	MGB_lrc38668_26638	1.4	20	0	No	ugauucacaugguucucgcc	−8.36
dka-miRN31	uaugcauaccgcuguaggacc	MGB_c17398_18804	2.2	98	0	Nes	gucuacagcggaauguauacc	−10.65
dka-miRN32	ugggaaagcuggcuuucgagg	MGB_s83401_48752	1.7	10	0	No	gagguggccaggauuuugca	−7.36
dka-miRN33	uuuauaaaguagaucucucgg	MGB_c33911_25155	1.7	148	0	No	aagggaucuacuuuacugaac	−11.25

### Validation of the miRNA expression by stem-loop qRT-PCR

**Figure 4:**
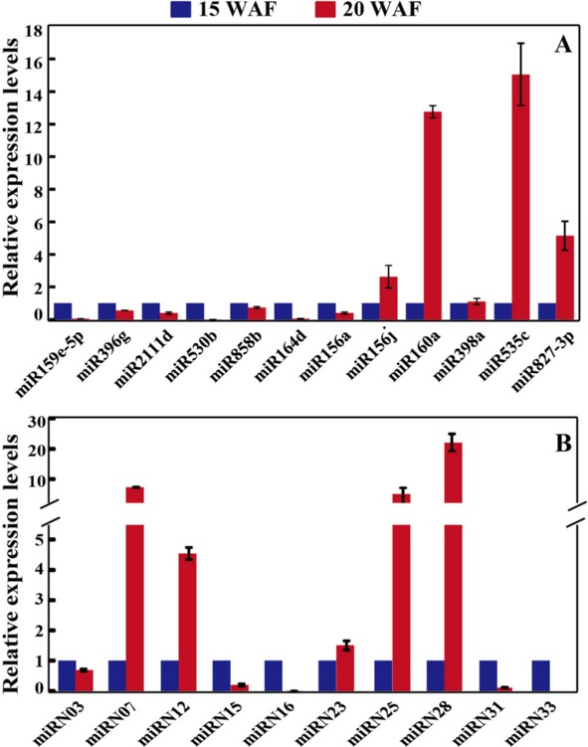
**Validation of the differentially expressed miRNAs identified by deep sequencing.** Expression analysis of known **(A)** and novel **(B)** miRNAs by stem loop qRT-PCR.

Stem-loop qRT-PCR, which is a reliable method for assessing miRNA expression levels, has been applied for experimental verification of the miRNAs [27,33]. For this purpose, we analyzed expression of 22 miRNAs, including 12 randomly selected known miRNAs (dka-miR159e-5p, dka-miR396g, dka-miR2111d, dka-miR530b, dka-miR858b, dka-miR164d, dka-miR156a, dka-miR156j, dka-miR160a, dka-miR398a, dka-miR535c, and dka-miR827-3p) and 10 novel miRNAs with relatively high expression levels (miRN03, miRN07, miRN12, miRN15, miRN16, miRN23, miRN25, miRN28, miRN31, and miRN33. The qRT-PCR analysis showed that expression patterns of the examined miRNAs (Figure 4A, B) were largely consistent with the results of deep sequencing except dka-miR156a, dka-miRN16, and dka-miRN28, which displayed opposite profiles between the two methods.

### Prediction of putative target genes for the known and novel miRNAs

Putative target genes for all of the known miRNAs were searched using psRNATarget. A total of 198 potential miRNA-target pairs were identified (Additional file 3: Table S2) from a transcriptome of ‘Eshi No. 1’ composed of 83,898 persimmon unigenes [10]. A number of the miRNAs have multiple targets, indicating the diversity of these miRNAs. The potential targets of known miRNAs were either conserved or non-conserved among different plants (Additional file 3: Table S2). Most of the predicted targets in this study were found to encode transcription factors (TFs). For example, miR156 was revealed to target squamosa promoter-binding protein-like (SPL) 9 and 5 of SPL family. Auxin response factor (ARF) 10 and 6 were found to serve as the targets of miR160. MiR319 was shown to target cycloidea and PCF transcription factor 3. In additions, several TFs in the families of basic helix-loop-helix (bHLH), growth-regulating factors (GRF), and MYB were predicted to act as targets of miR395p-3p, miR396d and miR858b, respectively. Interestingly, dka-miR396g targeted a gene encoding flavonoid 3-*O*-glucosyltransferase. In addition, some miRNAs targeted genes involve in disease resistance and stress response. For example, miR164 was found to target TIR class protein, while zinc finger (CCCH-type) family protein and copper/zinc superoxide dismutase were predicted targets of miR171 and miR398, respectively.

In addition, targets of the novel miRNAs were also predicted using the same strategy as that for the known miRNAs. 27 out of the 33 novel miRNAs can be successfully predicted to have their targets, which encode either transcription factors or functional genes that are involved in an array of processes, such as flower development, metabolism, and stress response (Additional file 3: Table S2). For example, miRN21 and miRN08 were predicted to target GRAS and AP2/B3-like TFs, respectively. Some target genes were shared by different novel miRNAs; for instance, NADH dehydrogenase was the target of miRN17 and miRN06. By contrast, a few novel miRNAs can target different genes; miRN32 was predicted to regulate plant invertase and pectin methylesterase inhibitor superfamily gene.

**Figure 5:**
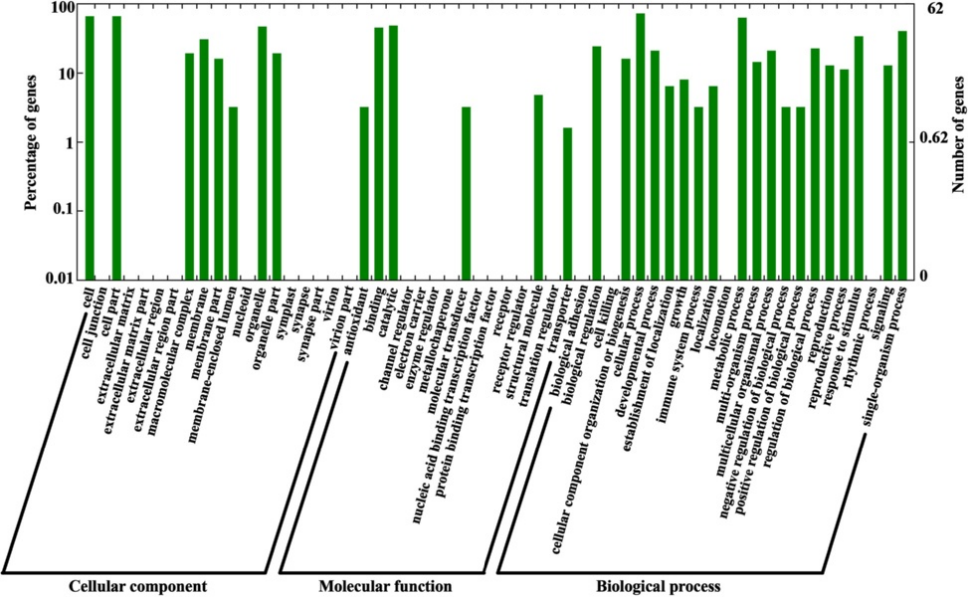
**Gene Ontology classifications of the target genes based on cellular component, molecular function, and biological process.**

To better understand regulatory roles of the identified miRNAs, we performed GO analyses on target genes of the differentially expressed known miRNAs (Additional file 4: Table S3). Among the 428 target genes, 246 were categorized into biological processes, 166 into cellular components and 16 into molecular functions (Figure 5). The major biological processes were ‘cellular process’ and ‘metabolic process’, such as GO:0016053 and GO:0009064. The main cellular components were ‘cell’ and ‘cell junction’, such as GO:0043232 and GO:0070013. As for molecular functions, the majority of genes were clustered into ‘binding proteins’ and ‘catalytic’, such as GO:0005524 and GO:0016407. The target genes regulated by the up-regulated miRNAs encode transcription factors (GO:0003700), whereas most of the targets regulated by the down-regulated miRNA were shown to take part in leaf development (GO:0048366), shoot system development (GO:0022621, GO:0048367), response to hormone stimulus (GO:0009725, GO:0032870), and hormone-mediated signaling (GO:0009755).

### Time-course expression dynamics of miRNAs and target genes

**Figure 6:**
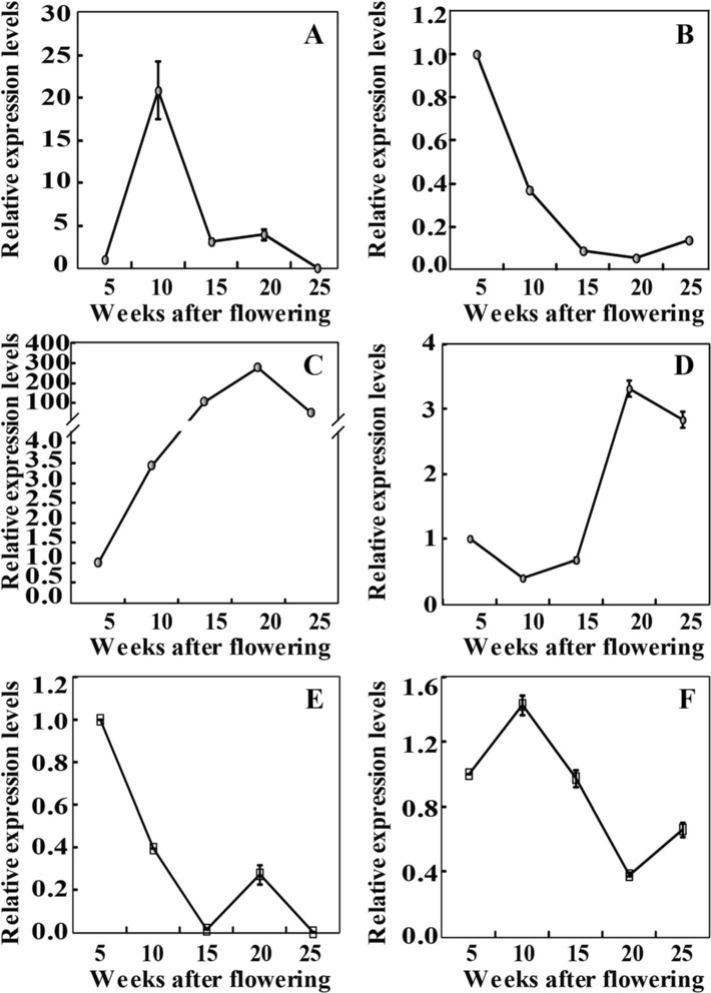
**Time-course expression analysis of four miRNAs and two target genes during fruit development of ‘Eshi No. 1’.** Expression patterns of miRNAs, including dka-miR156j-5p **(A)**, dka-miR858b **(B)**, dka-miR395p-3p **(C)**, and dka-miR2911a **(D)**, and target genes, including MGB_c41307 **(E)** and MGB_c15097 **(F)**, at the five stages, 5, 10, 15, 20 and 25 weeks after flowering (WAF).

In order to gain insight into the potential involvement of the miRNAs in (de)astringency, time-course expression profiles of four known miRNAs (dka-miR156j-5p, dka-miR858b, dka-miR395p-3p and dka-miR2911a) during fruit development were investigated using stem loop qRT-PCR. Transcript level of dka-miR156j-5p was very low at 5 WAF, sharply increased to the maximum value at 10 WAF, followed by a prominent decrease at 15 WAF. Then, the expression level was maintained constant at 20 WAF and decreased to undetectable level at the last time point (Figure 6A). As for dka-miR858b, the highest transcript level was detected at 5 WAF, which decreased continuously thereafter and reached the lowest level at 20 WAF, followed by a slight elevation at 25 WAF (Figure 6B). The mRNA abundance of dka-miR395p-3p began to accumulate at 10 WAF, and sharply increased by nearly 30 folds at 15 WAF, then progressively increased to the highest level at 20 WAF. At 25 WAF, the transcript level was reduced to the level of 15 WAF (Figure 6C). The mRNA abundance of dka-miR2911a was decreased to the minimum at 10 WAF, then slightly increased at 10 WAF, but remarkably increased to the highest expression level at 20 WAF, followed by a minor reduction at the last stage (Figure 6D).

In addition, expression profiles of MGB_c41307 (bHLH) and MGB_c15097 (alcohol dehydrogenase, ADH), two target genes regulated by dka-miR395p-3p and dka-miR2911a, respectively, were also analyzed using the same set of materials as mentioned above. Transcript level of MGB_c41307 was shown to be extremely high at the first stage of fruit development, but underwent a marked and steady decrease at 10 and 15 WAF, when the expression level was barely detected. MGB_c41307 was then up-regulated at 20 WAF, followed by a noticeable reduction at the last stage (Figure 6E). MGB_c15097 was induced from 5 to 10 WAF, but decreased steadily between 15 and 20 WAF, when the lowest expression level was observed. At the last time point, transcript level of MGB_c15097 was again elevated (Figure 6F).

### Verification of miRNA-guided cleavage of target genes by 5′-RACE

**Figure 7:**
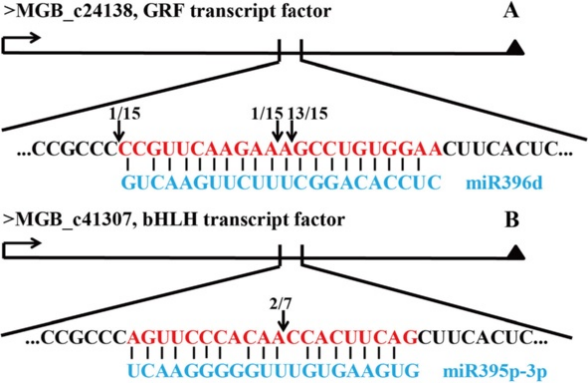
**Confirmation of two target genes by 5′-RLM-RACE.** Sequence comparison between the target genes, MGB_c24138 **(A)** and MGB_c41307 **(B)**, and their corresponding miRNAs, miR396d and miR395p-3p, respectively. The arrows indicate the cleavage sites, and the numbers show the frequency of clones sequenced.

Two target genes, MGB_c24138 (a GRF TF, target of mi396d) and MGB_c41307 (a bHLH TF, target of dka-miR395p-3p), were examined using 5′-RNA ligase-mediated RACE (5′-RLM-RACE) in order to confirm whether the target prediction was accurate. Two mismatches were observed between the amplified product of MGB_c24138 and mi396d. In addition, cleavage of MGB_c24138 primarily occurred at the tenth position of the miRNA sequence (Figure 7A). MGB_c41307 displayed three mismatches compared with the sequence of dka-miR395p-3p. Meanwhile, the cleavage was found to occur predominantly at the ninth position of the miRNA sequence (Figure 7B).

## Discussion

It has been well documented that miRNAs act as important regulatory factors that play pivotal role in a variety of biological processes, such as plant growth and development, morphogenesis, embryogenesis, stress tolerance, and metabolism [13,18,23,29]. Within the last years, enormous progresses have been made to explore miRNAs in several fruit trees, such as citrus, grape, apple, and peach [34-42]. However, the information concerning miRNA identification in persimmon is still limited compared to those identified in other fruit trees. Therefore, identification and characterization of miRNAs in persimmon will provide valuable knowledge for us to understand relevant regulation machinery pertinent to a specific process, such as (de)astringency.

In this study, we applied a Solexa sequencing platform for in-depth characterization of miRNAs in ‘Eshi No. 1’, an oriental persimmon that can lose astringency naturally on trees. About 13 million raw reads were obtained from the two libraries. The percentage of 24-nt sRNAs was much higher than that of 21-nt sRNAs. This finding is consistent with the results on tomato [43], but different from that of trifoliate orange [35], suggesting that distribution of sRNAs with different length may vary among plants. However, this may also be ascribed to sequencing or samples used for studies. As 24-nt sRNAs have been reported to function in heterochromatin modification, especially for genomes with a high content of repetitive sequences, the current study suggests that the sRNAs might play diverse roles in various processes of persimmon [44,45].

Based on the sequences available in the miRBase database and transcriptome data, 65 known miRNA families and 33 novel miRNAs have been identified from the libraries constructed using the persimmon fruits at two stages, when the soluble tannin underwent substantial reduction. Plant miRNA families have been classified into highly conserved, non-conserved and specific miRNAs [46]. In this study, we detected several highly conserved miRNA families, such as miR156, miR159/miR319, miR160 and miR166, which exhibited relatively abundant TPM in the persimmon libraries. This corroborates the proposition that these miRNAs serve as core miRNA families that are possibly ancestors of miRNAs in all land plants [45]. However, we also noticed that several other core miRNA members were not detected in this study, consistent with an earlier work with *Prunus* [47]. Three miRNA families, including miR477, miR482, and miR3627, have been reported to be highly conserved among fruit trees [48]; but we could only detect miR477 in persimmon. All of these findings seem to suggest that persimmon has not only conserved miRNAs as those previously reported, but also has its own set of miRNAs, which may be ascribed to the unique features of this plant in comparison with others.

In the current study, 61 out of the 236 known miRNAs were significantly differentially expressed in the fruits at the two examined stages, in which 17 were up-regulated at 20 WAF, and 44 were down-regulated. It is assumed that some, if not all, of the differentially expressed miRNAs might play key roles in regulating PA synthesis during persimmon fruit development. Interestingly, the differentially expressed miRNAs include several candidates, such as miR858 and miR156, that have been reported to regulate PA synthesis in other plants. MiR858 and miR156 have been reported to regulate biosynthesis of PAs in apple and *Arabidopsis thaliana*, respectively [41,49]. In our study, miR858 was down-regulated at 20 WAF, exhibiting a pattern similar to the change in soluble tannin content, implying that it may positively regulate the target genes involved in biosynthesis of PAs in persimmon. By contrast, miR156 was dramatically up-regulated (fold change >4) at 20 WAF, suggesting that it may act as a negative regulator of PA synthesis in persimmon. Meanwhile, miR395 might be another crucial miRNA for influencing PA synthesis as it is predicted to target genes associated with anthocyanin synthesis [10]. In addition, we also noticed that several differentially expressed miRNAs, such as miR398, miR399, and miR535, displayed dramatic fold changes between the two stages. Whether these miRNAs play roles in regulating PA synthesis remained to be investigated.

Bioinformatics comparison of sequence homology between miRNAs and gene sequences, a traditional approach for unraveling target genes in plants [50], demonstrated that 198 potential targets were successfully predicted for the miRNAs. Consistent with previous reports, a number of the targets in persimmon were also shown to encode transcription factors, including SPLs, ARFs, GRFs, bHLHs, and MYBs. Moreover, some target genes are shown to be involved in stress response, while others are implicated in a wide range of biological processes, implying a complexity of the miRNAs in the regulation of biological processes. We also noted that the novel miRNAs targeted different genes with a variety of functions, such as flower development (miRN21 and miR08), disease resistance (miRN28 and miRN33), and metabolism (miRN32) (Additional file 3: Table S2). All of these data suggest that the miRNAs are implicated in the regulation of genes associated with multiple processes during fruit development.

PAs are important secondary metabolites for determining the quality of persimmon fruits. PA biosynthesis shares the same upstream pathway as anthocyanins, both of which have been well characterized. Accumulating data suggest that regulation of the anthocyanin pathway largely depends on the interaction between R2R3 MYBs, bHLHs and WD40s [9,51,52]. This interaction may also play a role in the orchestration of the subset of genes associated with PA biosynthesis. In this regulatory machinery, MYB proteins may play a predominant role in activating the structural genes. Various members of R2R3 MYB family may exhibit different and separate regulatory impacts on the biosynthesis of end products in the flavonoid pathway branches leading to anthocyanins, flavonols, and proanthocyanins [53]. A number of earlier studies report that MYB genes might be regulated by various miRNAs [41,54,55]. Interestingly, in the current study we also detected a MYB-targeting miRNA, dka-miR858b, whose expression dynamics followed the same trend as the change in soluble tannins during fruit development, implying that dka-miR858b might be positively correlated with PA biosynthesis. This finding suggests that dka-miR858b may control a MYB protein that plays a negative role in regulating PA synthesis. This speculation is plausible as a few MYBs acting as repressors have been reported in earlier studies, such as *FaMYB1* of strawberry and *VvMYB4* of grape, although most MYBs behave as positive regulators to govern the expression of structural genes [56,57]. However, more work is needed to characterize the MYB gene regulated by dka-miR858b. Apart from MYB, we also found that a bHLH protein was predicted to be targeted by dka-miR395p-3p, which was experimentally verified using 5′-RLM-RACE. The expression pattern of dka-miR395p-3p was precisely opposite to that of the bHLH gene, providing another line of evidence supporting the reverse regulation. Moreover, expression profile of the bHLH gene was largely concordant with the fluctuation of tannin, except the last time point, suggesting that dka-miR395p-3p/bHLH regulation might be involved in the regulation of PA synthesis. SPL has been documented to act as one of the miR156 targets in earlier work [49]. In our study, we discovered a differentially expression miRNA, dka-miR156j-5p, which was predicted to target SPL. Strikingly, dka-miR156j-5p was down-regulated to extremely low abundance with the ripening of fruits (15–25 WAF), implying that SPL might be abundantly expressed during the late stages of fruit development. SPL has been reported to destabilize the MYB-bHLH-WD40 complex, which in turn leads to repression of anthocyanin biosynthetic genes and inhibits anthocyanin accumulation [58]. In this regard, it is assumed that the stability of MYB-bHLH-WD40 complex might be increasingly impaired with the extension of fruit development. This causes an adverse effect on the activation of the structural genes pertinent to the flavonoid pathway, leading to lowered PAs biosynthesis.

It has been documented that the metabolites derived from different enzymatic reactions form trans- or *cis*-flavan-3-ol units, such as 2, 3-trans-(+)-catechin and 2,3- *cis*-(−)-epicatechin [2], which are the precursors of PAs. They are generally glucosylated by 3-*O*-glucosyltransferase, thus facilitating transportation to vacuole and polymerization [11]. Therefore, the gene/enzyme responsible for glucosylation is critical for PAs biosynthesis. In the study, dka-miR396g was revealed to target flavonoid 3-*O*-glucosyltransferase gene. Transcript abundance of dka-miR396g was down-regulated in the fruits at 20 WAF in comparison with those of 15 WAF. This implies that expression of 3-*O*-glucosyltransferase gene might be enhanced when fruits ripened, which in turn leads to accelerated glucosylation and facilitates the transportation of precursors.

In this study, the soluble tannin was decreased with the ripening of persimmon fruits, whereas the insoluble counterpart was increased. Growing evidences demonstrate that PAs insolubility is a critical factor responsible for loss of astringency in persimmon fruits [28]. Conversion from soluble into insoluble tannin, especially at the late stage of fruit development, requires the participation of acetaldehyde, which is formed *in situ* from ethanol with a catalytic reaction mediated by alcohol dehydrogenase (ADH) and pyruvate decarboxylase [8,59]. Interestingly, an ADH-targeting miRNA, dka-miR2911a, was acquired in the sequencing data. Of note, expression pattern of dka-miR2911a was perfectly inverse to that of the ADH gene, which, however, exhibited a trend same as the change in the insoluble tannin. Therefore, we speculate that dka-miR2911a played an instrumental role in promoting insolubility of tannin by regulating ADH gene at the late stage of fruit development.

## Conclusion

**Figure 8:**
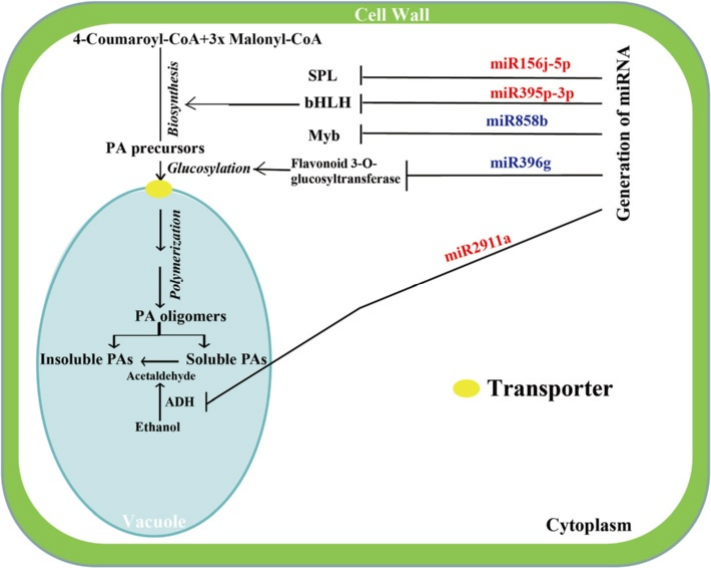
**A hypothetical model of action indicating the involvement of identified miRNAs in PAs biosynthesis and deastringency of persimmon fruits.** The miRNAs in red are up-regulated at 20 weeks after flowering (WAF), whereas those in blue are down-regulated.

In this study, through high throughput sequencing we identified 236 known miRNAs belonging to 65 miRNA families, and 33 novel miRNAs in persimmon. Of the identified known miRNAs, 61 were shown to be differentially expressed during fruit development, 17 up-regulated and 44 down-regulated. Some of the differentially expressed miRNAs were predicted to target an array of genes that have been previously reported to be involved in synthesis, transportation and insolubility of PAs, all of which are limiting factors associated with PA accumulation. Based on our data and earlier studies, we propose a model on deastringency of CPCNA by integrating the identified miRNAs in the PAs biosynthesis pathway (Figure 8). Of the miRNAs, miR395p-3p and miR858b regulate bHLH and MYB, which work in synergy to regulate the structural genes responsible for PA biosynthesis. However, towards the fruit ripening, miR156j-5p/SPL regulates the stabilization of MYB-bHLH-WD40 complex, leading to decreased PAs production. However, transportation of the PAs to vacuole is expedited when PA precursors were glycosylated by flavonoid 3-*O*-glucosyltransferase, which is targeted by miR396g. Finally, soluble tannin was converted to insoluble part in the vacuole through acetaldehyde produced from ethanol oxidization mediated by ADH, which is under the control of miR2911a, in particular at the late stage of fruit development. The identification of these miRNAs paves way for elucidating the post-transcriptional regulation of (de)astringency in persimmon. In the future, extra work is required to functionally characterize the identified miRNAs and verification of their direct targets that are tightly involved in the regulation of PAs accumulation and deastringency of CPCNA.

## Methods

### Plant materials

‘Eshi No. 1’ (*D. kaki*) planted at the Persimmon Repository of Huazhong Agricultural University (30° 289′ N, 114° 219′ E) was use in this study. Fruits were harvested at 5, 10, 15, 20, and 25 weeks after flowering (WAF). Three different trees were used, and 10 representative fruits from each tree were collected. The fruits were peeled, and the pulp at the equatorial part was collected, immediately frozen in liquid nitrogen and maintained at −80°C until use.

### Determination of PA using printing and folin-ciocalteu method

Soluble tannins were detected by an imprinting method using FeCl_2_, which reacts with soluble tannin to form blue-black tannin-iron compounds [60]. For this purpose, filter papers were immersed in 5% FeCl_2_ aqueous solution for 15–30 min, then dried at 50–60°C. Cross sections prepared from equatorial parts of the fruits were printed on the filter papers and kept still for about 5 min, followed by visual observation of the color change (Darker printing indicates presence of more tannin in the pulps).

In addition, quantitative determination of soluble tannin and insoluble tannin contents was performed using folin-ciocalteu method [61]. About 5 g of frozen samples was ground into powder in liquid nitrogen and extracted with 15 ml of 80% methanol (80: 20, v/v). The homogenate was centrifuged for 10 min at 5000 × *g*; the supernatants containing soluble tannins were transferred to 50-ml measuring cylinders. The procedure was repeated twice. The supernatants were combined and adjusted to a final volume of 50 ml using distilled water, and used as working solutions. For measurement of soluble tannins, 1 ml of the working solution was mixed with 7.5 ml distilled H_2_O, and then 0.5 ml of 2 N folin-ciocalteu reagents (Sigma) was added. After a thorough vortex, the mixture was maintained still for 3 min at room temperature, to which 1 ml of saturated Na_2_CO_3_ was added. After incubation for 1 h at room temperature, the solution was measured on a spectrophotometer (UV-1800, Shimadzu, Japan) by reading absorbance at 725 nm. The residues were then used to extract insoluble tannins. They were suspended in 15 ml of 1% HCl-methanol (1:99, v/v) and placed at room temperature for 30 min, after which same manipulations were employed as those for measuring soluble tannin. Soluble and insoluble tannins were expressed as tannin acid equivalents per gram of fresh weight (FW).

### Small RNA library construction and sequencing

Total RNA was extracted from the pulp using RNA plant Plus (Tiangen) according to the manufacturer’s instructions. Purity and integrity of the RNA was checked using agarose gel electrophoresis and quantified by determining the absorbance at 260 nm. Three μg of the total RNA was used for preparation of small RNA libraries with Balancer NGS Library Preparation Kit for small/microRNA (GnomeGen). Briefly, the RNA was ligated to 3′ and 5′ adaptors sequentially, reverse transcribed to cDNA and amplified using PCR. The library was applied to a 10% native PAGE gel, and bands corresponding to miRNA insertion were cut and eluted. After ethanol precipitation and washing, the purified small RNA libraries were quantified with Qubit Fluorometer (Invitrogen) and used for cluster generation and 36 nt single-end sequencing analysis with Illumina GAIIx (Illumina, San Diego, CA, USA). The small RNA libraries sequencing was completed in ABLife company (Wuhan, China).

### Bioinformatics analysis of the sequenced miRNAs

After the sequencing was completed, raw sequences were pre-processed using the Fastx-toolkit pipeline, which included removing the low-quality tags and trimming adaptor sequences to generate high quality reads (or clean reads). The trimmed sequences were used for searching against Rfam (11.0) database using BLASTN program to exclude known non-coding RNAs, including rRNAs, tRNAs, small nuclear RNAs (snRNAs) and small nucleolar RNAs (snoRNAs). The remaining sequences were then searched against the miRBase v19.0 database [62] to identify putative known miRNAs, which were then subjected to analysis of length distribution, nucleotide preference at each position and the first nucleotide preference.

Small RNAs that were not mapped to any pre-miRNAs in the miRBase or not classified into any categories in the Rfam (11.0) database were subsequently analyzed against the persimmon transcriptome data using miRDeep2 [63,64] to check their potential as novel miRNAs. Sequences with perfect matches were then used to predict secondary structures by Mfold 3.2 [65]. The main criteria used for selecting potential precursors include: production of hairpin structures containing mature miRNA sequences from one arm and miRNA^*^ from the opposite one, maintenance of compatible minimum free energy and stability, and presence of two nucleotides at the 3′ overhangs in the miRNA/miRNA^*^ duplex [66].

### Differential expression analysis of the miRNAs

To determine expression patterns of miRNAs between 15 and 20 WAF, the frequency of miRNA counts was normalized as transcripts per million (TPM). Normalization of miRNA expression levels between the two samples was carried out based on actual miRNA count/total count of clean reads × 10^6^. Fold change in the miRNAs between 15 and 20 WAF was then determined from the normalized data using the formula ‘fold change = log_2_ (20 WAF /15 WAF)’.

Differential expression analysis was calculated using the software edgeR [67]. Fold change and *P*-value were combined to determine the significance of final miRNA expression, and the expression difference was considered as significant if |fold change| >1 and *P*-value < 0.05. A positive value indicated up-regulation of a miRNA at 20 WAF in comparison with 15 WAF, while a negative value indicated down-regulation.

### Target gene prediction and functional annotation

Target genes of the differentially expressed miRNAs were bioinformatically predicted based on degree of sequence homology between miRNAs and targets [68]. In this study, target gene prediction was performed by analysis on psRNAtarget program [69] and WMD3 (http://wmd3.weigelworld.org/cgi-bin/webapp.cgi) using the persimmon ESTs and transcriptome database (MGB.Unigene.seq [10]) as described earlier. Gene Ontology (GO) enrichment was used to analyze function and biological processes of the target genes. GO terms significantly enriched in the target genes were compared to the reference gene background. Three major GO categories were analyzed, including cellular component, biological process, and molecular function [70].

### Analysis of miRNA expression by stem-loop qRT-PCR

Stem-looped qRT-PCR was used to validate the differentially expressed miRNAs and to analyze time-course expression of four miRNAs at five developmental stages [27,33]. Stem-loop primers (Additional file 5: Table S4) were designed according to Chen et al. [71]. Total RNA was treated with gDNA Eraser (TaKaRa, Dalian, China) to remove genomic DNA according to the manufacturer’s instructions. RNA was then reverse-transcribed into cDNA with specific stem-loop primers in a 20-μl reaction buffer composed of 4 μl of 5 × PrimeScript ® Buffer 2, 1 μl of PrimeScript® RT Enzyme Mix, 4 μl of RNase-free dH_2_O. The qRT-PCR was performed on a LightCycler® 480 Real-Time System (Roche, Switzerland). Each PCR reaction, in a total volume of 10 μl, contained 5 μl of 2 × QuantiFast SYBR Green PCR Master Mix (QIAGEN), 200 ng of cDNA, 0.35 μl of forward primer and universal reverse primer. Reactions were performed at 95°C for 5 min, and 95°C for 10 s, 61°C for 30s, 72°C for 10 s for 45 cycles, and a melting temperature cycle with constant fluorescence data acquisition from 65 to 95°C. Ubiquitin 6 was used as an internal control for miRNAs. Each sample was analyzed in four replicates, and the qRT-PCR data were analyzed with LightCycler® 480 software version 1.5 (Roche).

### Analysis of target gene expression by quantitative RT-PCR

Expression of two target genes was assayed with qRT-PCR according to the protocols mentioned above except using non-stem loop primers for reverse transcription and gene-specific primers for qRT-PCR. The primers were designed using Primer 5.0 software based on the gene sequences. Actin was used as an internal control for normalizing the target gene expression, and the procedures were operated in the same manner as those of miRNAs.

### 5′-RNA ligase-mediated rapid amplification cDNA ends

5′-RNA ligase-mediated RACE (RLM-RACE) was performed with the GeneRacer kit (Invitrogen) as described previously [72]. First, 10 μg of total RNA was ligated to the 5′-RACE RNA Oligo adaptor. The ligated mRNA was reverse transcribed into cDNA using oligo (dT) primer. To obtain the 5-terminus products, PCR was performed using 5′ GeneRacer™ primer and gene-specific primers (Additional file 5: Table S4). The PCR products were cloned, sequenced and subjected to analysis of the cleavage site on target mRNAs by corresponding miRNAs.

## Availability of supporting data

The sRNA data reported in this work are deposited in the database of NCBI (National Center for Biotechnology Information), under the accession number of SRP050516. The information can be accessed via the link http://www.ncbi.nlm.nih.gov/bioproject/PRJNA269202. The information of small RNA libraries at the two stages can be accessed via the links http://www.ncbi.nlm.nih.gov/sra/?term=SRX796050 (15 weeks after flowering, WAF) and http://www.ncbi.nlm.nih.gov/sra/?term=SRX796055 (20 WAF), respectively.

## Additional files

Additional file 1: Table S1. Analysis of identified known miRNA and miRNA family. Additional file 2: Figure S1. Identification of novel miRNAs. Additional file 3: Table S2. Target prediction of known and novel differentially expressed miRNAs. Additional file 4: Table S3. GO annotation of the putative targets regulated by the differentially expressed miRNAs. Additional file 5: Table S4. Primers and their sequences used in this study.

## Abbreviations

ADH: Alcohol dehydrogenase
bHLH: Basic helix–loop–helix
CPCNA: Chinese pollination constant non-astringent
FW: Fresh weight
JPCNA: Japanese pollination constant non-astringent
miRNAs: microRNAs
nt: Nucleotide
PAs: Proanthocyanidins
PCNA: Pollination constant non-astringent
qRT-PCR: Quantitative real-time reverse transcription polymerase chain reaction
RACE: Rapid amplification of cDNA ends
TF: Transcription factor
TPM: Transcripts per million
WAF: Weeks after flowering
