# Supplementary material for: Identification and characterization of microRNAs from Chinese pollination constant non-astringent persimmon using high-throughput sequencing
Source: BMC Plant Biol. 2015 Jan 21;15:11. doi: 10.1186/s12870-014-0400-6 (PMC4308916; doi:10.1186/s12870-014-0400-6)
Supplement: Additional file 3: Table S2. — Target prediction of known and novel differentially expressed miRNAs. [file 12870_2014_400_MOESM3_ESM.xlsx]

# Gene Expression Variability in Chronic Lymphocytic Leukemia

**Simone Ecker*, Vera Pancaldi*, Daniel Rico† & Alfonso Valencia†**

## Additional File 1

## Supplementary Figures
## Supplementary Tables
## Supplementary Methods

# Supplementary Figures

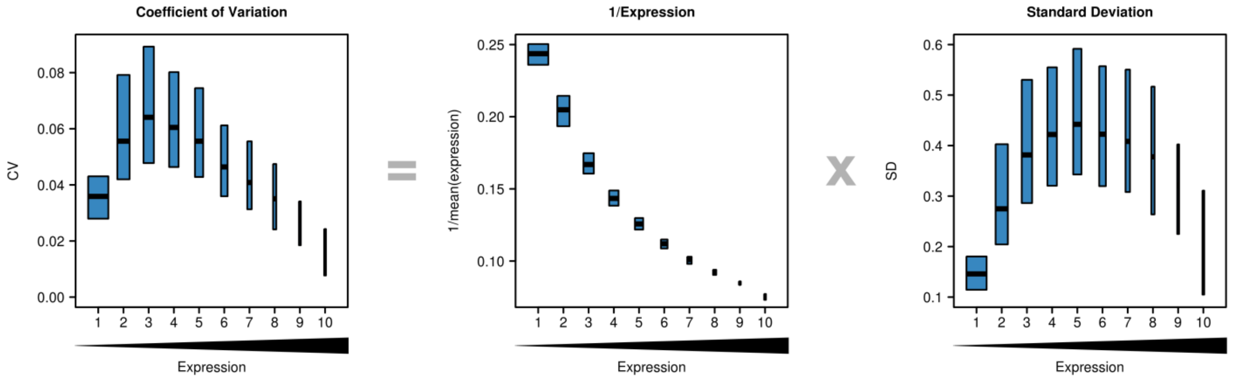


**Supplementary Figure 1: Definition of the Coefficient of Variation (CV) and its dependence on gene expression levels.** Left panel: CV versus expression of genes in bins of increasing expression level (see Supplementary Methods and Supplementary Table 3). Middle panel: Relationship between reciprocal of mean expression and expression in bins of increasing expression level. Right panel: Dependence of the standard deviation of expression across patients on the level of expression.

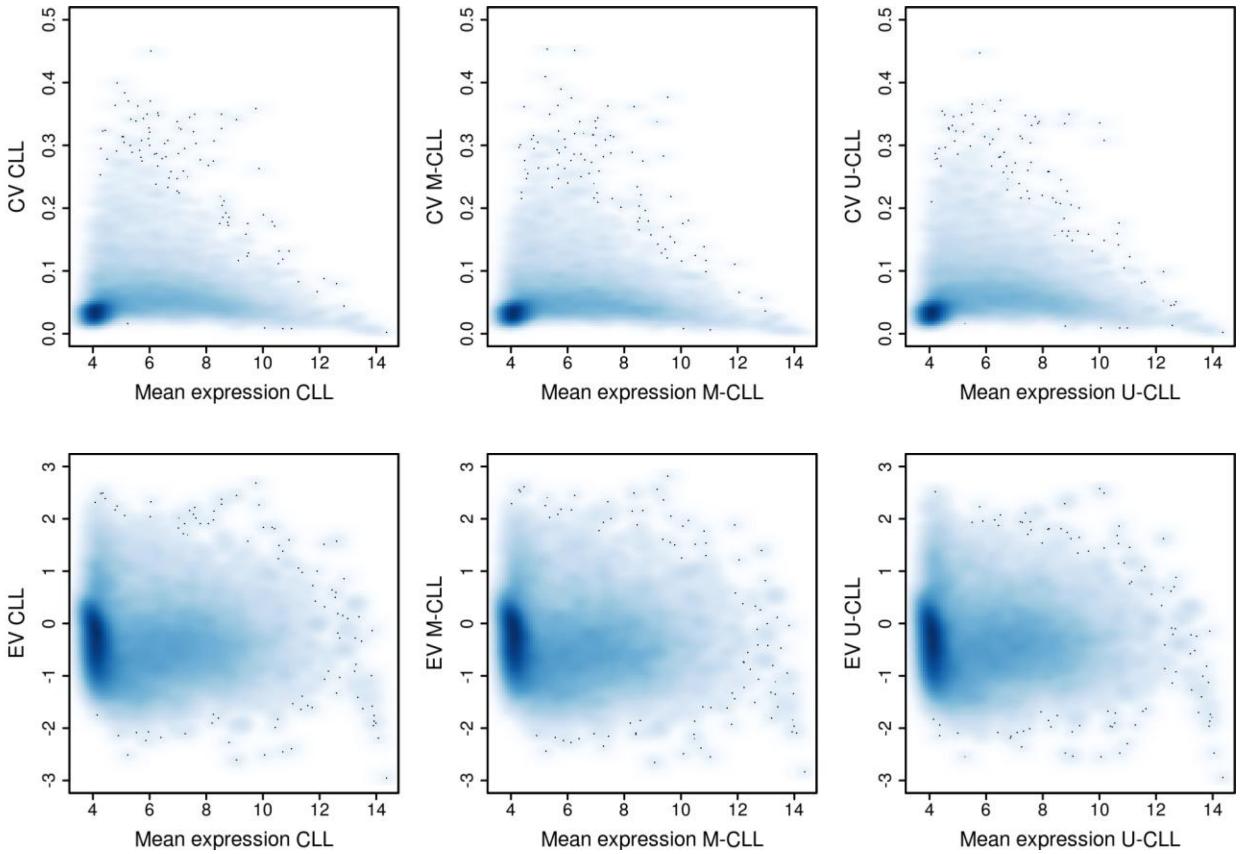


**Supplementary Figure 2: Scatterplots of CV and EV distributions and their correlation with mean expression.** Darker colors indicate an increased density of data points in the corresponding region of the plot. Top row: CV versus mean expression across all CLL samples, and across only M-CLL and U-CLL samples respectively. Bottom row: The same for the EV.

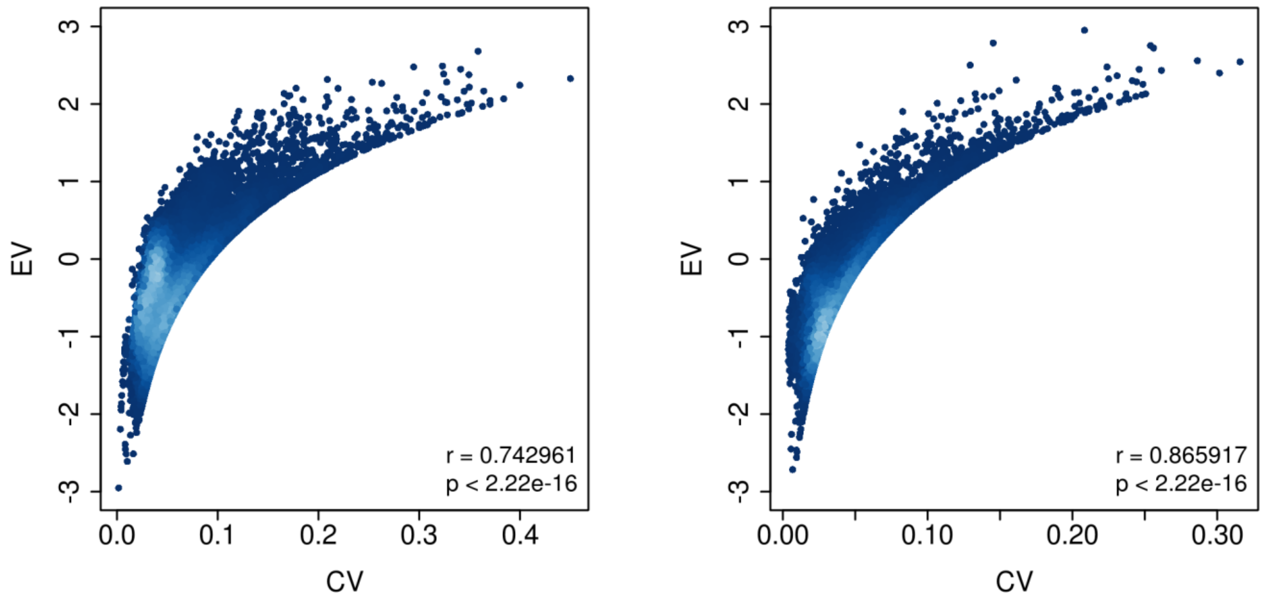


**Supplementary Figure 3: Correlation of CV and EV in Kulis et al. and Fabris et al. data.** Lighter colors indicate higher densities of data points in the corresponding regions of the plot. Left panel: Scatterplot of CV versus EV using the Kulis et al. dataset. Right panel: Scatterplot of CV versus EV using the Fabris et al. dataset.

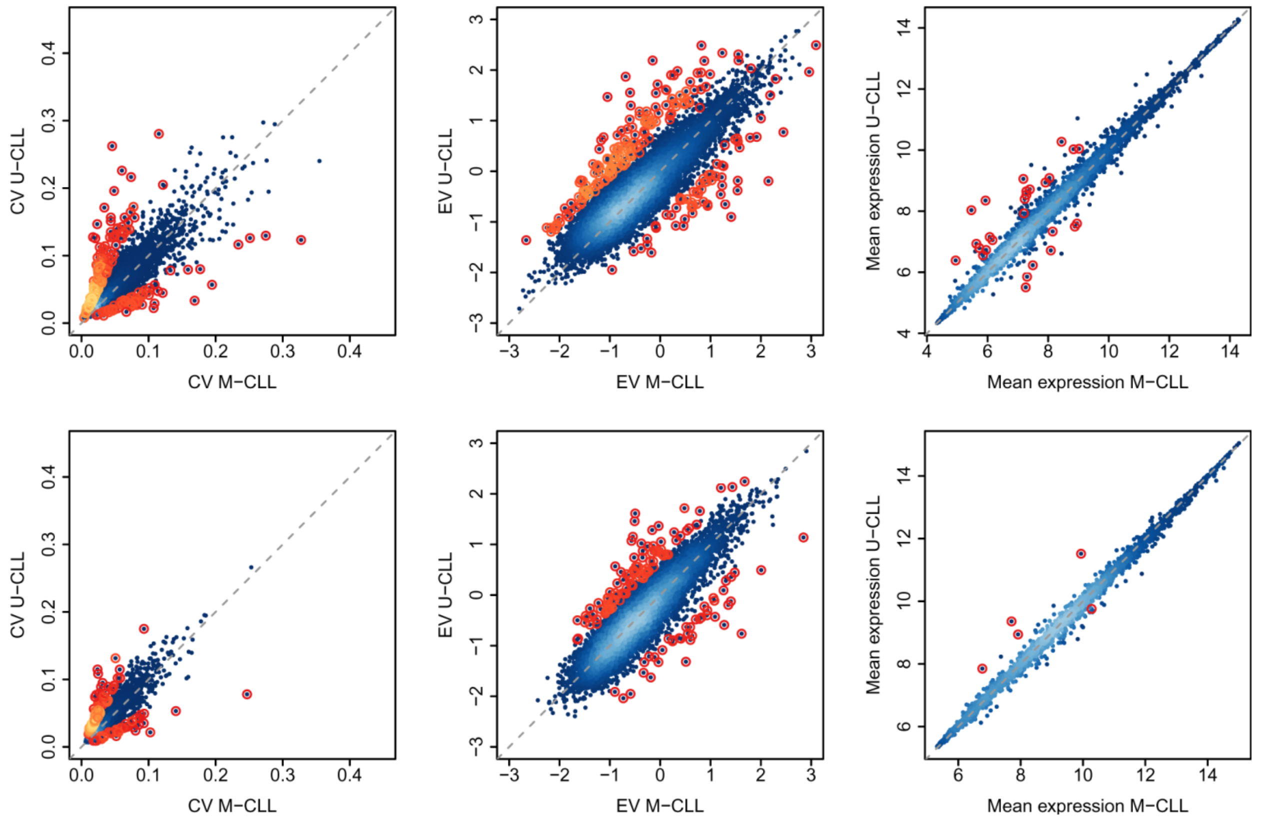


**Supplementary Figure 4: Gene expression variability comparison of M-CLL and U-CLL in Fabris et al. and Haslinger et al. data.** Lighter colors indicate higher densities of data points in the corresponding regions of the plot. Genes with statistically significant p-values at an FDR of 5% are highlighted. The gray dashed line represents the identity line. Left panel: Scatterplot of CV across patients in the two disease subtypes. Genes with statistically significant differential variability according to the F-test are highlighted. Middle panel: Scatterplot of EV across patients in the two disease subtypes. Genes with statistically significant differential variability according to the F-test are highlighted again. Right panel: Scatterplot of mean expression levels across patients in the two disease subtypes. Genes with statistically significant differential expression are highlighted. Top row: Data of Fabris et al. Bottom row: Data of Haslinger et al.

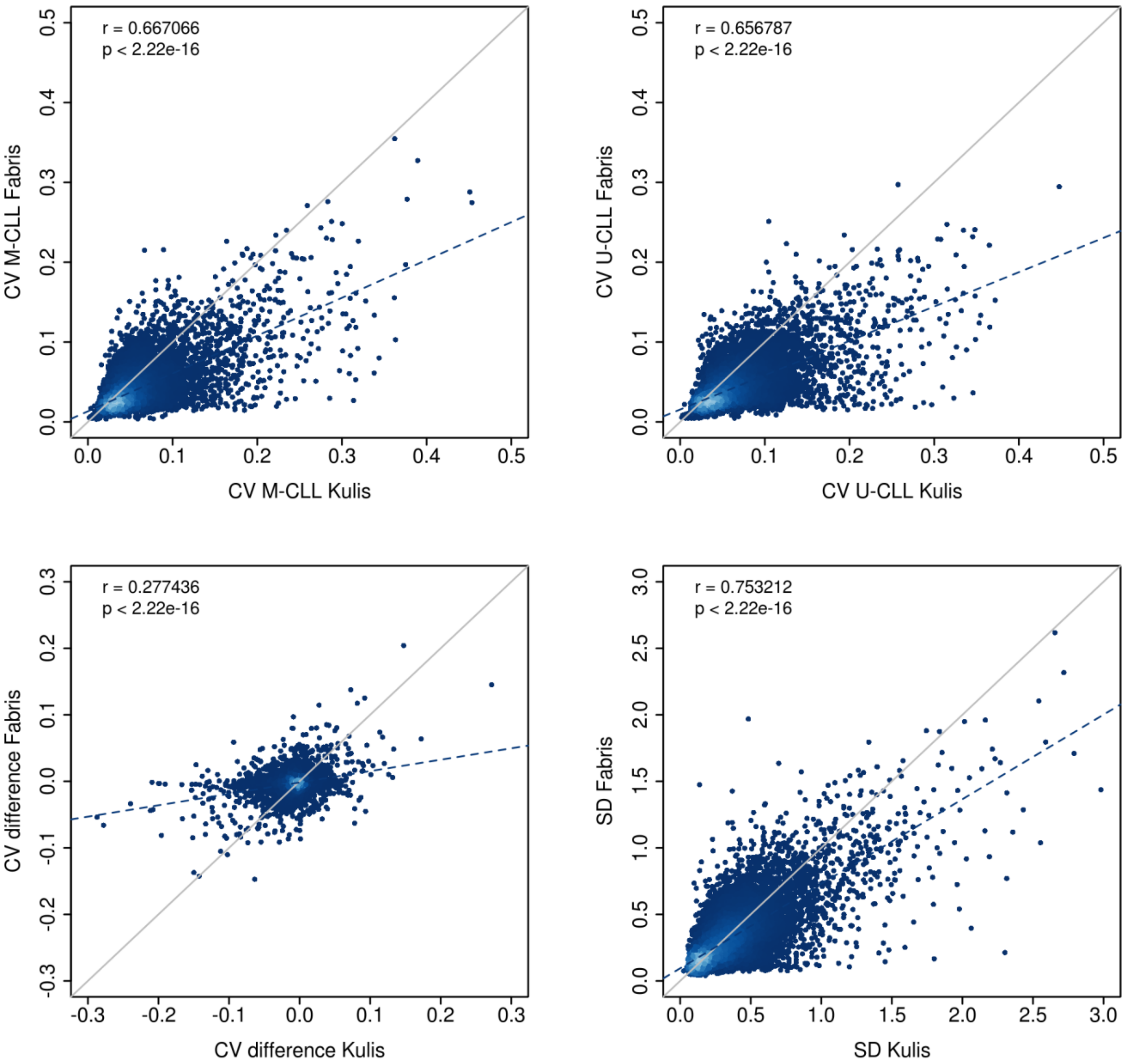


**Supplementary Figure 5: Correlation of variability measurements between the data of Kulis et al. and Fabris et al.** Scatterplots comparing Kulis et al. and Fabris et al. data. Lighter colors indicate higher densities of data points in the corresponding regions of the plot. The gray line represents the identity line, the blue dashed line represents the fitted regression line. Upper left panel: CV of M-CLL in Kulis versus CV of M-CLL in Fabris. Upper right panel: CV of U-CLL in Kulis versus CV of M-CLL in Fabris. Lower left panel: CV difference ($CV_{M\text{-}CLL}$ - $CV_{U\text{-}CLL}$) in Kulis versus CV difference in Fabris. Lower right panel: Standard deviation across all CLL samples in Kulis versus standard deviation in Fabris.

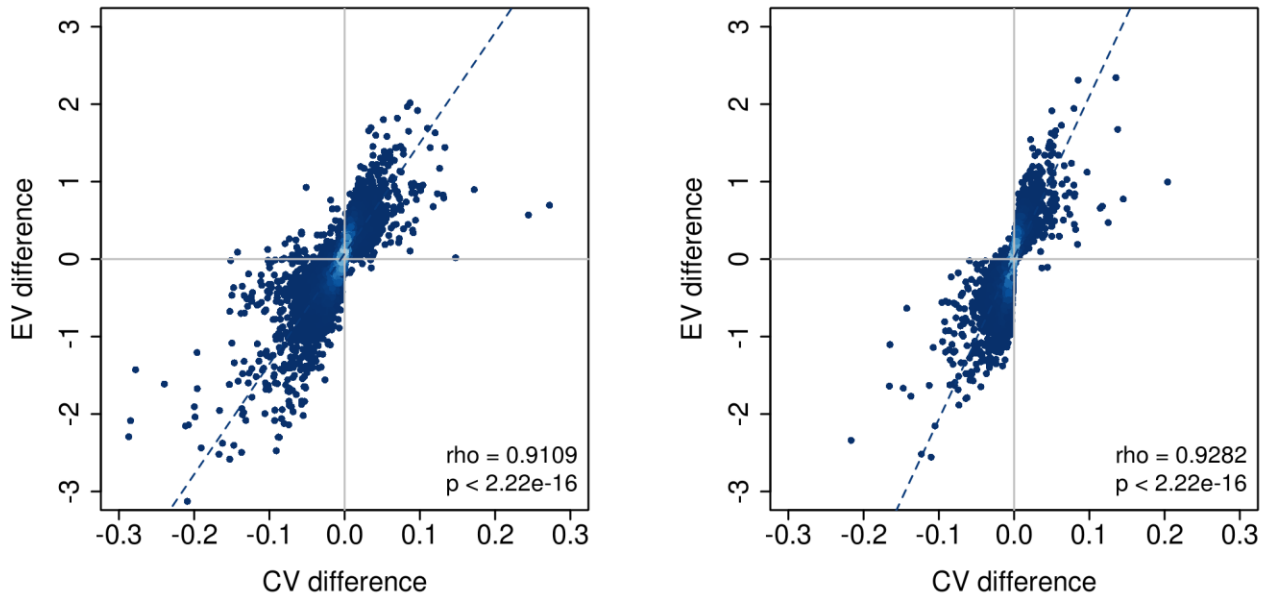


**Supplementary Figure 6: Correlation of CV difference and EV difference in Kulis et al. and Fabris et al. data.** CV difference = $CV_{M\text{-}CLL}$ - $CV_{U\text{-}CLL}$ and EV difference = $EV_{M\text{-}CLL}$ - $EV_{U\text{-}CLL}$. Lighter colors indicate higher densities of data points in the corresponding regions of the plot. The blue dashed line represents the fitted regression line. Left panel: Scatterplot of CV difference versus EV difference using the Kulis et al. dataset. Right panel: Scatterplot of CV difference versus EV difference using the Fabris et al. dataset.

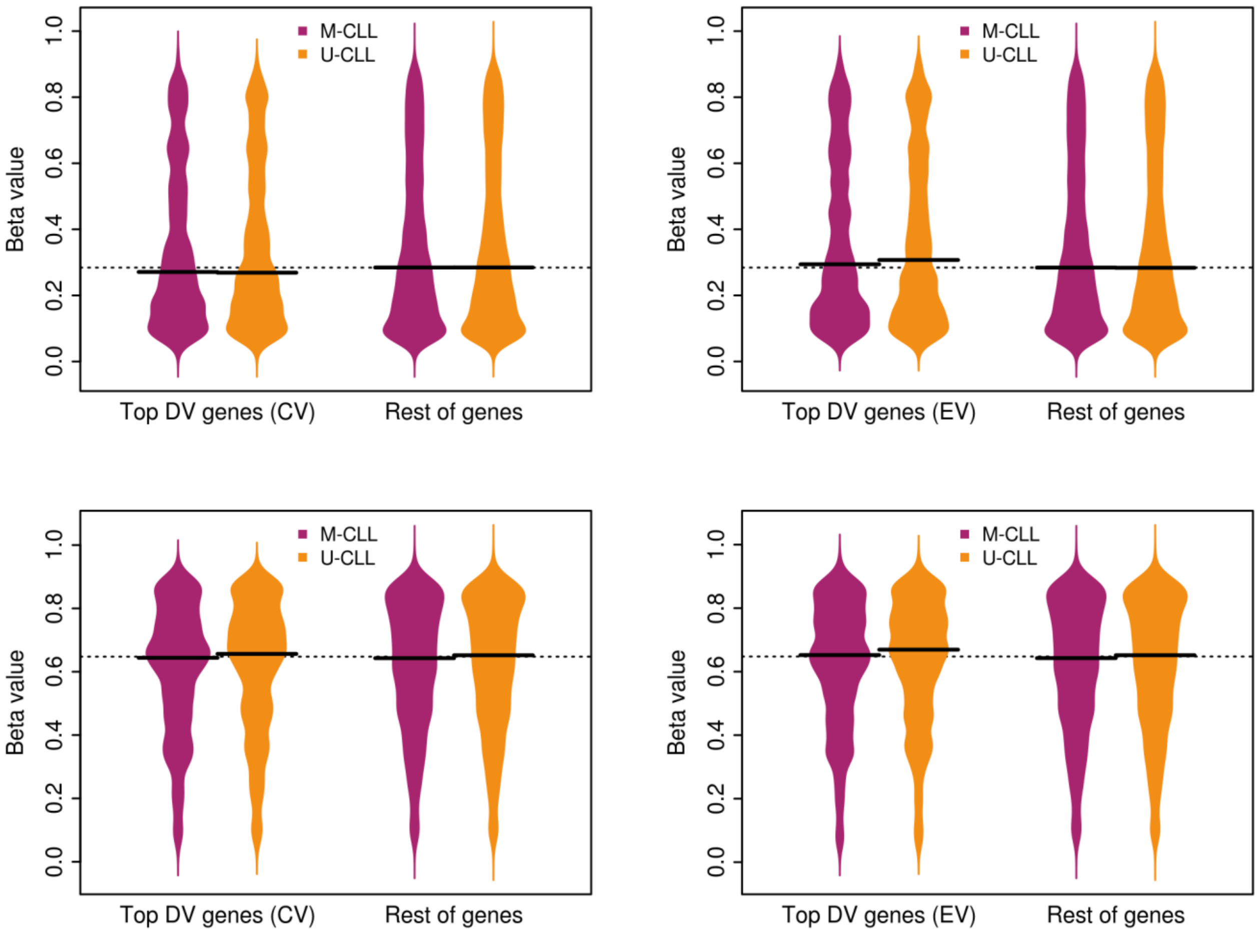


**Supplementary Figure 7: Beanplots comparing the methylation profiles of M-CLL and U-CLL of the top 500 genes with increased variability in U-CLL.** Methylation measurements are given in beta values. Top row: Promoter methylation. Bottom row: Gene body methylation. Left panel: Differential variability based on CV differences. Right panel: Differential variability based on EV differences.

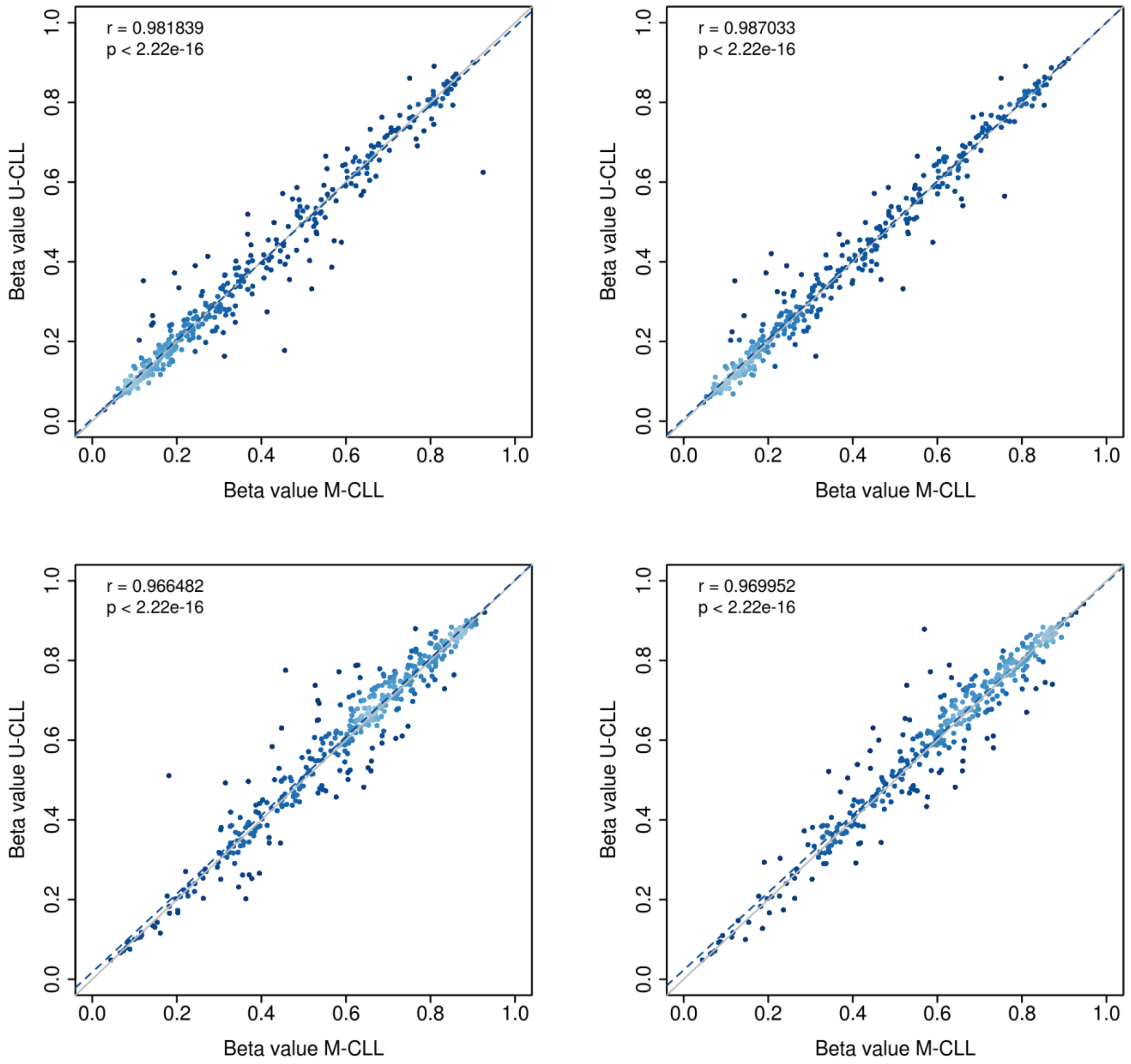


**Supplementary Figure 8: Correlation of methylation values of the top 500 genes with increased variability in U-CLL.** Scatterplots comparing M-CLL and U-CLL. Lighter colors indicate higher densities of data points in the corresponding regions of the plot. The gray line represents the identity line, the blue dashed line represents the fitted regression line. Top row: Promoter methylation. Bottom row: Gene body methylation. Left panel: Gene list obtained from differential variability based on CV differences. Right panel: Gene list obtained from differential variability based on EV differences.

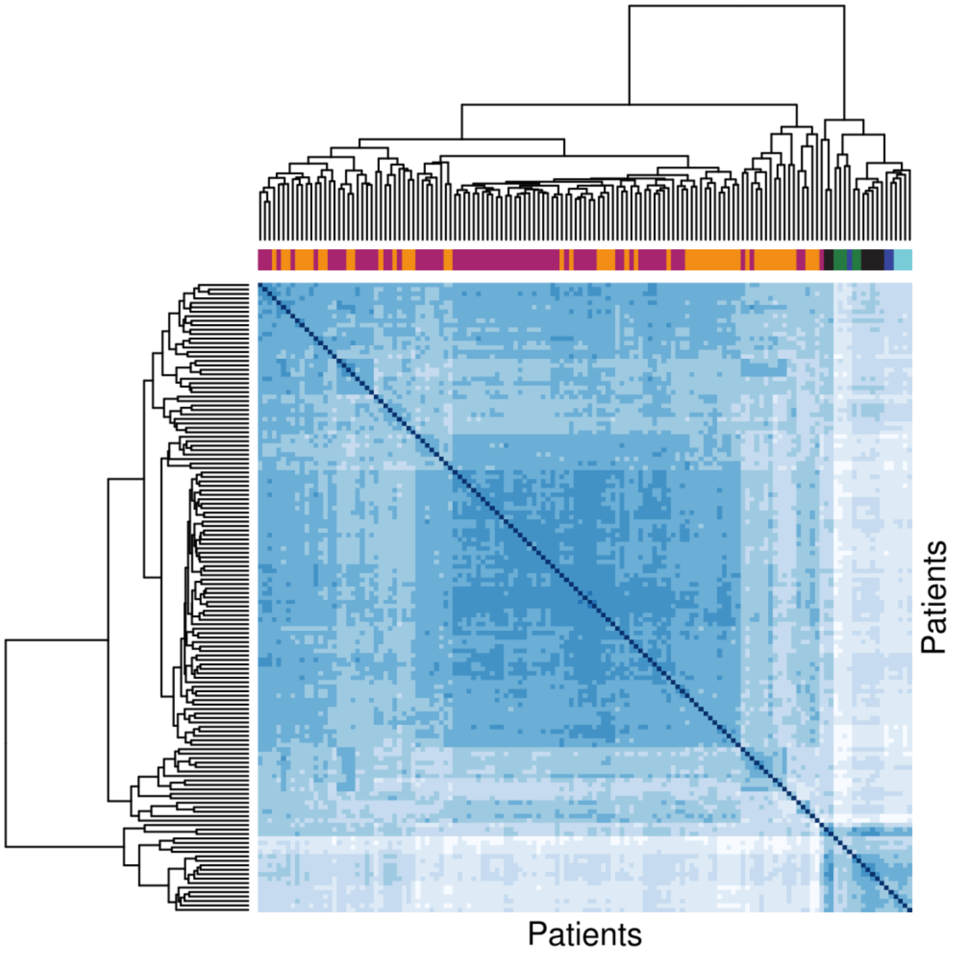


**Supplementary Figure 9: Hierarchical clustering of gene expression data.** Heatmap representing the clustering of the CLL patients of the study of Kulis et al. U-CLL samples are colored in orange, M-CLL samples in dark magenta, and healthy cells in blue, green and black.

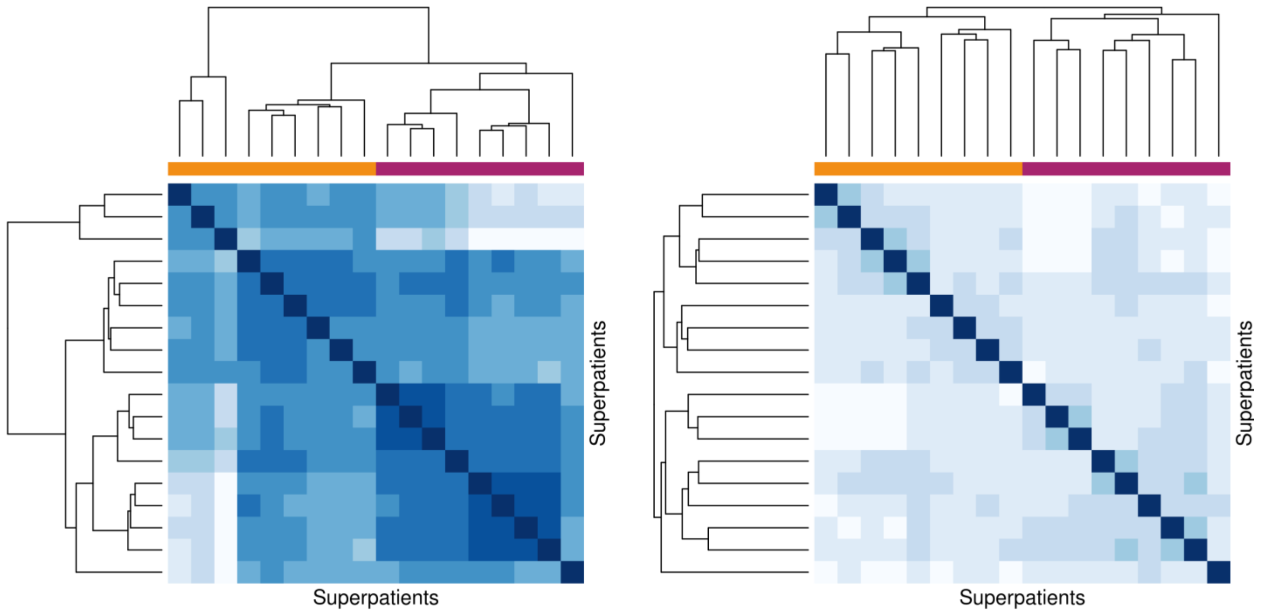


**Supplementary Figure 10: Hierarchical clustering of superpatients** (see Supplementary Methods for the definition of ‘superpatients’). Heatmaps representing the clustering of superpatients into U-CLL and M-CLL, based on aggregate measures (see Supplementary Methods). Left panel: Superpatient clustering based on mean expression values. Right panel: Superpatient clustering based on the CV. Results very similar to the figure on the right were obtained when using other measurements of variability such as the EV, SD, IQR or different distance measures (data not shown). The figures shown here are examples chosen arbitrarily. As the superpatient approach relies on random subsampling of the patients, the results are different after every new run of patient aggregation, therefore the results of the hierarchical clusterings vary as well.

# Supplementary Tables

**Supplementary Table 1: Results of F-tests in the three datasets analyzed.**
Column all: all genes. Column sig: genes with statistically significant p-values (F-test, FDR=0.05).

| Dataset | Increased variability in M-CLL | | Increased variability in U-CLL | |
|---|---|---|---|---|
| | all | sig | all | sig |
| ICGC | 6,425 | 360 | 13,871 | 2,025 |
| Fabris | 4,936 | 64 | 9,793 | 172 |
| Haslinger | 3,829 | 44 | 6,459 | 106 |

**Supplementary Table 2: Results of Hypergeometric tests assessing the overlap between genes within differentially methylated regions and genes with increased expression variability in U-CLL.** Column Number of genes: The number of genes for which their promoters or gene bodies have been identified to lie within differentially methylated regions (see Methods). Column Overlap DV genes: The number of the genes reported in the previous column which are also contained in the list of the top 500 genes with increased variability in U-CLL for which methylation measurements are available (n=491). Column p-value: p-value of the Hypergeometric test evaluating if the overlap between the gene lists is bigger than expected by chance.

| Region | Hypermethylated in M-CLL | | | Hypermethylated in U-CLL | | |
|---|---|---|---|---|---|---|
| | Number of genes | Overlap DV genes | p-value | Number of genes | Overlap DV genes | p-value |
| Promoter | 381 | 15 | 0.2647 | 238 | 8 | 0.5174 |
| Gene body | 165 | 5 | 0.6296 | 173 | 6 | 0.4989 |

**Supplementary Table 3: Gene expression bins.**

| Bin number | Expression values | Nr of genes in bin |
|---|---|---|
| 1 | < 4.5 | 9,101 |
| 2 | ≥ 4.5 and < 5.5 | 3,355 |
| 3 | ≥ 5.5 and < 6.5 | 2,402 |
| 4 | ≥ 6.5 and < 7.5 | 1,987 |
| 5 | ≥ 7.5 and < 8.5 | 1,517 |
| 6 | ≥ 8.5 and < 9.5 | 986 |
| 7 | ≥ 9.5 and < 10.5 | 435 |
| 8 | ≥ 10.5 and < 11.5 | 224 |
| 9 | ≥ 11.5 and < 12.5 | 74 |
| 10 | ≥ 12.5 | 68 |

# Supplementary Methods

### Gene expression bins

We established 10 classes of gene expression levels from lowly to highly expressed genes.
The overall expression values range from 3.6 to 14.4. See Supplementary Table 2.

### The ‘superpatient’ approach of aggregating patients

We aggregated patients into groups by extracting 5 random U-CLL patients and another 5 M-CLL patients as long as sets of 5 could be made without repeating the patients in the groups. For each random group of 5 patients we calculated the mean expression and the expression CV. We thus produced a new cohort of ‘superpatients’ of which half are U-CLL and the other half M-CLL. These superpatients represent constructs that help to de-noise the data and to obtain surrogates of variability values. Changing the number of patients used to create the superpatients like for example taking 7 or 10 random samples instead of 5 did not alter the results.

### Hierarchical clustering

Hierarchical agglomerative clustering was performed on the Spearman correlation matrix of the gene expression and the methylation dataset from Kulis et al. using the function Agnes from package cluster in R using the ‘average’ method and default settings for the distance metric. Heatmaps were then generated using heatmap.2 from the package gplots.
